# Development of fuzzy-based ranking alternatives by hybrid RSM-fuzzy AHP-fuzzy CoCoSo framework sustainable ultrasonic-assisted turning of magnesium alloys

**DOI:** 10.1038/s41598-025-32693-5

**Published:** 2026-01-09

**Authors:** S. P. Sundar Singh Sivam, V. G. Umasekar, K. Stalin, Adebayo Samuel Olabisi

**Affiliations:** 1 Department of Mechanical Engineering, College of Engineering and Technology, SRM Institute of Science and Technology, Kattankulathur, 603203 Tamil Nadu India; 2 Department of Marine Engineering, Amet University, East Coast Road, Kanathur, Chennai, 603 112 India; 3 Mechanical Engineering Department, William VS Tubman University, Harper, Liberia

**Keywords:** UAT, Stir casting, Magnesium alloys, Optimization, Response surface methodology (RSM), Fuzzy AHP, Fuzzy CoCoSo, Sustainable manufacturing, Engineering, Materials science, Mathematics and computing

## Abstract

This study aims to optimize the machining performance of magnesium alloys fabricated by stir casting in ultrasonic-assisted turning (UAT) by enhancing surface quality, extending tool life, and increasing material removal rate (MMR), contributing to sustainable manufacturing practices. A hybrid optimization approach combining Response Surface Methodology (RSM) and Fuzzy CoCoSo was employed. The RSM design, based on the Hybrid Box-Behnken model, was used to select experimental parameters, while Fuzzy AHP determined fuzzy criteria weights. The optimal process parameters were identified by Fuzzy CoCoSo. The study focused on cutting speed (11.00 to 42.00 m/min), feed rate (0.0500 to 0.1500 mm/rev), and ultrasonic power intensity (0.0000 to 100.00%). A total of 15 runs in a quadratic design were conducted to assess surface roughness, tool wear, MMR, energy consumption, and dimensional accuracy. Confirmation experiments validated the model’s predictions. The study revealed that ultrasonic parameters, particularly frequency and amplitude, significantly influenced machining performance. The optimal settings of 26.5 m/min cutting speed, 0.15 mm/rev feed rate, and 100% ultrasonic power intensity resulted in improved surface roughness (0.72 μm), enhanced MMR (0.95 cm³/min), reduced tool wear (60 μm), lower energy consumption (160 W), and better dimensional accuracy (97%). Although the study provides valuable insights into UAT for stir-cast magnesium alloys, further investigations are needed to evaluate the long-term effects of optimized parameters and their applicability to different alloy compositions. The optimized parameters can be directly applied in industrial machining of stir-cast magnesium alloys, offering both cost-efficiency and sustainability benefits, including reduced energy consumption, improved surface quality, and longer tool life. This research introduces a novel hybrid optimization framework integrating RSM, Fuzzy AHP, and Fuzzy CoCoSo to optimize UAT for stir-cast magnesium alloys, advancing sustainable machining technologies.

## Introduction

Ultrasonic-assisted turning (UAT) is a modern machining approach where high-frequency vibrations are applied to the cutting tool, improving its efficiency in machining hard-to-machine materials. It offers notable advantages such as reduced cutting forces, lower tool wear, and enhanced surface finish, making it attractive for precision-driven industries. However, the effect of vibration parameters on machining performance remains insufficiently understood. This study aims to optimize UAT parameters to extend tool life, improve surface quality, and enhance energy efficiency, thereby promoting sustainable and cost-effective manufacturing.

The study of magnesium alloys, particularly their fabrication processes, microstructure, mechanical properties, and applications, has seen extensive contributions. Ahmadi et al. (2022)^1^ reviewed selective laser melting (SLM) of magnesium alloys, emphasizing its advantages in producing high-performance components, discussing its challenges like defects and issues in microstructure and mechanical properties. Antoniac et al. (2022)^2^ focused on the use of magnesium alloys in orthopedic surgery, presenting their potential for medical implants, and the challenges they face regarding corrosion resistance and biocompatibility. Chen et al. (2020)^3^ investigated the incorporation of zoledronate into magnesium alloys for orthopedic applications, addressing the improvement in corrosion resistance and bioactivity. Ding et al. (2022)^4^ studied the anticorrosion and tribological properties of AZ31 magnesium alloys, demonstrating that Nb2O5 coatings enhanced their durability and wear resistance. Homma et al. (2009)^5^ proposed a high-strength magnesium alloy produced through hot extrusion, showing significant improvements in its mechanical properties. Kishore et al. (2022)^6^ explored AZ31B magnesium alloy for microfabrication, specifically micro-EDM, highlighting its potential in producing intricate micro products. Meher et al. (2020)^7^ investigated the effect of TiB2 reinforcement on the mechanical and microstructural properties of magnesium RZ5 alloy-based composites, improving its strength and wear resistance. Meng et al. (2023)^8^ fabricated Zn2+-loaded polydopamine coatings on magnesium alloy surfaces to enhance corrosion resistance and biocompatibility. Mohanavel et al. (2021)^9^ reviewed the mechanical properties of titanium diboride-reinforced aluminum matrix composites, shedding light on their relevance for advanced manufacturing processes. She et al. (2012)^10^ developed a superhydrophobic CuO surface on AZ91D magnesium alloys, which significantly improved corrosion resistance. Wang et al. (2022)^11^ applied superhydrophobic Zn-Ni coatings to LA43M magnesium alloys to further enhance their performance in harsh environments. Wu et al. (2023)^12^ reviewed recent advancements in grain refinement of magnesium rare-earth alloys during casting, discussing methods to achieve fine grains for better performance. Yuan et al. (2016)^13^ developed a biodegradable magnesium alloy-based esophageal stent via coating with elastic polymer, improving its potential as a medical implant. Zhang et al. (2015)^14^ worked on the fabrication of superhydrophobic surfaces on magnesium alloys, further enhancing their corrosion resistance. Zhao et al. (2022)^15^ presented mechanisms of ultrafine-grain fabrication in AZ31 magnesium alloys using squeeze casting-extrusion, which significantly affected their mechanical properties.

The machinability of magnesium alloys has been an area of significant interest due to their widespread use in aerospace, automotive, and biomedical applications. Arunachalam et al. (2024)^16^ investigated the machining process of MoS2/B4C/AZ31 magnesium alloy composites, focusing on their tribological characteristics during machining, and proposed ways to enhance the machining performance. Berzosa et al. (2019)^17^ conducted a feasibility study on hole repair and maintenance operations of magnesium alloys in aeronautical components, specifically focusing on dry drilling. They found that optimization of the drilling process improved the hole repair efficiency and quality. Berzosa et al. (2020)^18^ extended this work by geometric optimization of drills used in magnesium alloys, focusing on improving the performance and precision of hole repairs in magnesium components. Deswal and Kant (2022)^19^ examined the synergistic effect of ultrasonic vibration and laser energy during hybrid turning operations on magnesium alloys, highlighting the benefits in reducing surface roughness and improving tool life. Juraimi et al. (2020)^20^ reviewed the performance enhancement of energy saving and machining characteristics in electrical discharge machining (EDM) of magnesium alloys, concluding that optimization of EDM parameters could enhance the machining efficiency. Khanna et al. (2019)^21^ studied the effect of hybrid machining techniques on magnesium PMMC (particle-matrix composites), identifying the optimal machining parameters for improved mechanical properties. Mostafapor and Vahedi (2019)^22^ investigated the effects of process input parameters on the performance of wire EDM in AZ91 magnesium alloys, showing that the selection of appropriate parameters led to improved surface quality and material removal rate. Okokpujie et al. (2022)^23^ focused on the experimental study of nano-lubricants in temperature reduction during machining of Al-Si-Mg composite using the Deform 3D FEM method, showcasing their positive impact on temperature control. Pu et al. (2014)^24^ modeled the microstructural changes during dry and cryogenic machining of AZ31B magnesium alloys, indicating that cryogenic machining improved material properties compared to conventional methods. Rao and Srinivas (2022)^25^ optimized laser micro machining on magnesium alloy AS21-SiC composites using empirical models, contributing to sustainable machining practices. Saeed et al. (2024)^26^ conducted a comparative study on sustainable machining for magnesium alloys using GRA and TOPSIS methodologies, demonstrating their effectiveness in optimizing process parameters. Samuel et al. (2021)^27^ reviewed the effect of machining on aluminum alloys with a focus on the 6061 alloy, providing insights into the processing challenges and advancements. Stalin et al. (2020)^28^ explored ultrasonic machining of tellurium copper metal matrix composites, emphasizing the effectiveness of ultrasonic vibrations in improving surface integrity. Sundar Singh Sivam et al. (2018)^29^ optimized input parameters for better product quality in the machining of magnesium AM60 using grey relation analysis and ANOVA. Sundar Singh Sivam et al. (2019)^30^ further enhanced the drilling process parameters for AM60 magnesium alloys, focusing on improving quality characteristics using the Taguchi-ranking algorithm.

Ultrasonic-assisted machining (UAM) has emerged as a promising technique for improving the machining of hard-to-cut materials like magnesium alloys. Shareef et al. (2022)^31^ analyzed the effect of rotary ultrasonic machining parameters on the surface integrity of advanced ceramics, providing insights into the benefits of ultrasonic vibrations in enhancing machining performance. Sharma and Pandey (2016a)^32^ optimized machining and vibration parameters for minimizing residual stresses in UAT of 4340 hardened steel, emphasizing its applicability in improving the surface quality of magnesium alloys as well. Sharma and Pandey (2016b)^33^ reviewed recent advances in ultrasonic-assisted turning (UAT), emphasizing its potential to enhance machining efficiency and sustainability. Sharma and Pandey (2018)^34^ experimentally investigated surface roughness during UAT with self-lubricating cutting inserts, highlighting the benefits of this approach in reducing tool wear. Sharma and Pandey (2019)^35^ developed a mechanistic cutting force model for UAT with self-lubricating cutting inserts, further improving the understanding of UAT’s advantages in machining magnesium alloys. Singh et al. (2023)^36^ provided a comprehensive review of rotary ultrasonic machining of advanced materials, exploring the effectiveness of ultrasonic vibration in improving surface finish and reducing tool wear during machining of magnesium alloys. Singh and Khamba (2006)^37^ reviewed ultrasonic machining of titanium and its alloys, drawing parallels to magnesium alloys and suggesting ways to optimize their machining through ultrasonic techniques. Sofuoğlu et al. (2018a)^38^ conducted experimental investigations of machining characteristics and chatter stability for Hastelloy-X with ultrasonic and hot turning, providing insights into the application of UAT in improving the machining process for magnesium alloys. Sofuoğlu et al. (2018b)^39^ numerically investigated hot UAT of aviation alloys, providing recommendations for improving machining precision. Soleimanimehr (2021)^40^ analyzed the cutting ratio’s influence on diametrical errors in UAT, which can be applied to improve machining of magnesium alloys. Tong et al. (2012)^41^ studied the residual stress of GCr15 bearing steel during ultrasonic vibration turning, offering valuable insights into how ultrasonic vibration affects the material properties of magnesium alloys. Umasekar and John (2024)^42^ investigated UAT of magnesium silicon carbide composites, providing experimental insights into the effectiveness of ultrasonic machining for these advanced materials. Umasekar and Stalin John (2023)^43^ conducted experimental investigations on AISI D2 tool steel during ultrasonic-assisted turning, contributing to the growing body of knowledge on UAT’s impact on magnesium alloys. Venkata Sivareddy et al. (2022)^44^ studied the effect of thermo-mechanical loading on machining-induced residual stresses in ultrasonic vibration-assisted turning of Ti6Al4V alloy, which is relevant for magnesium alloys as well. Wang et al. (2016)^45^ investigated surface grinding of CFRP composites using rotary ultrasonic machining, providing insights into the application of ultrasonic machining for improving the surface finish of magnesium alloys. Wang et al. (2020)^46^ studied the effects of elliptical ultrasonic vibration on surface machining of CFRP composites, suggesting similar improvements for magnesium alloys. Wang et al. (2018)^47^ reviewed the damage formation and suppression in rotary ultrasonic machining of hard and brittle materials, offering guidelines for optimizing UAT on magnesium alloys. Wei et al. (2022)^48^ studied surface roughness models in 3D ultrasonic vibration-assisted turning, which could be applied to enhance the machining of magnesium alloys. Wei et al. (2023)^49^ conducted theoretical and experimental studies of 3D ultrasonic vibration-assisted turning, providing new insights into improving the machining of magnesium alloys. Xu et al. (2019)^50^ experimentally studied chip formation in UAT of 304 stainless steel, drawing parallels for improving chip formation in magnesium alloy machining. Xu et al. (2021)^51^ studied the cutting forces in UAT of 304 stainless steel, contributing valuable data on force dynamics that can be applied to magnesium alloys. Xu et al. (2023)^52^ investigated tool wear in UAT of 304 stainless steel, which has implications for the longevity of tools used in magnesium alloy machining. Ya et al. (2002)^53^ analyzed the ultrasonic machining mechanism, providing insights into the underlying physics that can be applied to enhance UAT for magnesium alloys. Yu et al. (2004)^54^ studied 3D micro-ultrasonic machining, which is relevant for precise machining of magnesium alloys. Yuan et al. (2023)^55^ reviewed the advances in ultrasonic vibration machining of SiCp/Al composites, suggesting similar approaches for enhancing the machining of magnesium alloys. Zamani et al. (2021)^56^ investigated UAT’s employment in microtextures to improve surface adhesion of titanium implants, a process that can be adopted for magnesium alloys. Zhang et al. (2019)^57^ developed an electromechanical dynamics model of ultrasonic transducers, which can be applied to improve the performance of UAT on magnesium alloys. Zhang et al. (2023)^58^ studied single-excitation 3D ultrasonic turning technology, providing insights into optimizing UAT for magnesium alloys. Zhang et al. (2023)^59^ designed a high-speed rotary ultrasonic machining tool for brittle materials, offering methods to improve UAT’s effectiveness for magnesium alloys. Zhou et al. (2019)^60^ reviewed advances in rotary ultrasonic machining systems for hard and brittle materials, providing valuable methods for improving the machining of magnesium alloys. Zou et al. (2017)^61^ studied diamond tool wear in UAT of die steels, offering lessons that could enhance the longevity of tools used in magnesium alloy machining. Zou et al. (2015)^62^ conducted experiments on ultrasonic vibration-assisted turning of 304 stainless steel, providing data that can be used to optimize UAT on magnesium alloys.

Fuzzy multi-criteria decision analysis (MCDA) methods have seen significant contributions in decision-making processes, particularly in evaluating complex systems. Feizizadeh et al. (2014)^63^ introduced a GIS-based fuzzy MCDA for landslide susceptibility mapping, which could be applied to the evaluation of materials or processes in manufacturing. Figueiredo et al. (2021)^64^ proposed a Life Cycle Sustainability Assessment framework based on BIM and fuzzy AHP, which can be used to evaluate the sustainability of materials like magnesium alloys in construction. Frazão et al. (2018)^65^ conducted a systematic review of MCDA in healthcare, providing insights into its application in evaluating different manufacturing techniques for magnesium alloys. Frini (2017)^66^ applied fuzzy sets theory and artificial intelligence in MCDA, contributing to more nuanced decision-making in manufacturing processes. Gigović et al. (2016)^67^ developed a GIS-fuzzy DEMATEL MCDA model for evaluating ecotourism sites, which can be adapted for evaluating materials and processes in manufacturing. Indrajayanthan et al. (2022)^68^ used a SWOT-MCDA methodology to explore energy transition scenarios in India, which can be used to evaluate the environmental impact of magnesium alloy production. Ouma et al. (2014)^69^ applied fuzzy AHP and GIS in optimizing urban highway bypass alignment, which could be adapted for the optimization of process parameters in magnesium alloy machining. Thakur et al. (2022)^70^ utilized fuzzy entropy and complex proportional assessment for group decision-making, which could be applied in selecting optimal machining methods for magnesium alloys. Yatsalo et al. (2021)^71^ extended the PROMETHEE method for fuzzy decision-making, providing a methodology for evaluating different fabrication methods for magnesium alloys. Ziemba (2021)^72^ used fuzzy MCDA to select electric vehicles, offering an approach that can be used to evaluate sustainable manufacturing practices for magnesium alloys.Sha et al. (2025)^73^ developed the ZHPO-LightXBoost model for predicting pesticide residues in crops from small datasets using an integrated optimization and boosting framework; results showed improved prediction accuracy and robustness, suggesting future expansion to larger agro-data and real-time applications. Sha et al. (2025)^74^ proposed an automatic 3D reconstruction method for transparent objects combining multiple optimization strategies under limited constraints, achieving higher surface fidelity and reduced error, with future work directed toward multi-view integration. Ma et al. (2025)^75^ introduced a topology optimization method for thermal-fluid problems in motorized spindle cooling jackets to enhance heat dissipation efficiency; numerical results validated rapid convergence, with future studies targeting multi-physics applications. Liu and Shen (2025)^76^ designed an AI-based system for exercise pressure measurement to improve data acquisition accuracy, reporting high real-time performance and suggesting integration with wearable health devices. Liu et al. (2025)^77^ developed a compensator-based fixed-time control for vehicular platoons with input nonlinearities, enhancing stability and response time; future research will address scalability in autonomous networks. Li et al. (2025)^78^ modeled relative fatigue life of marine helical gears under 3D mixed lubrication, finding load-dependent life reduction and recommending micro-geometry optimization. Ma et al. (2025)^79^ proposed a grooved heat-pipe method for spindle system thermal control at low speed, achieving superior temperature uniformity and efficiency; future work includes design scaling for industrial motors. Ni et al. (2025)^80^ performed numerical analysis of ultrasonic spot welding of Cu/Cu joints to optimize bonding strength and temperature distribution, suggesting further parametric validation. Xu et al. (2024)^81^ investigated fuel injection stability in marine low-speed dual-fuel engines, improving combustion uniformity through optimized timing control and proposing real-time adaptive control in future engines. Liu et al. (2025)^82^ examined granite fracture behavior under true triaxial stress, finding that lateral stress significantly affects shear strength and crack propagation; future tests will explore temperature effects. Laghari et al. (2019)^83^ reviewed soft computing techniques in machining of particle-reinforced metal matrix composites, highlighting optimization via AI and recommending hybrid intelligent systems for future development. Ali et al. (2022)^84^ optimized turning parameters for SiCp/Al 45 wt% composites to reduce cutting forces using DOE and regression analysis, achieving minimal force values and proposing multi-objective optimization. Laghari and Sarhan (2025)^85^ modeled machinability performance of 50% SiCp/Al MMCs using ANFIS and MRA, achieving accurate prediction of surface quality and wear, with future focus on hybrid optimization. Laghari et al. (2023)^86^ studied milling behavior of SiCp/Al MMCs under sustainable cooling, showing improved tribological properties and surface finish; future research targets eco-fluid design. Laghari et al. (2024)^87^ applied genetic modeling to optimize machining of high volume fraction SiCp/Al composites, enhancing performance and reducing tool wear, with future work on real-time adaptive genetic algorithms.

The novelty of this work lies in the integration of fuzzy logic techniques with traditional optimization methods, such as Response Surface Methodology (RSM), to optimize the machining of magnesium alloys using ultrasonic-assisted turning (UAT). Unlike conventional optimization methods like Taguchi, genetic algorithms, and artificial neural networks (ANN), which either rely on fixed assumptions or require extensive data with limited interpretability, the hybrid RSM-Fuzzy AHP-Fuzzy CoCoSo approach provides a more comprehensive and adaptable solution.

Traditional methods, such as Taguchi and genetic algorithms, primarily focus on minimizing variation by optimizing a set of predefined parameters based on fixed assumptions. While these methods are effective for certain applications, they often do not account for the complexity and interdependence of multiple factors that influence machining performance. ANN, though capable of handling non-linearities, typically requires large datasets and can lack clarity regarding how specific parameters impact the machining process. In contrast, the RSM-Fuzzy AHP-Fuzzy CoCoSo model addresses these shortcomings by integrating expert judgment through Fuzzy AHP, enabling the inclusion of subjective evaluation in a systematic framework. The Fuzzy CoCoSo method further enhances this approach by providing a decision-oriented ranking of alternatives based on weighted criteria, thus offering a more holistic solution that accounts for conflicting objectives such as surface finish, tool wear, material removal rate (MMR), and energy consumption.

A key limitation in the existing literature is the insufficient understanding of how UAT parameters affect magnesium alloys, especially those fabricated by stir casting. Previous studies have often lacked a comprehensive optimization framework that balances critical machining factors, leaving gaps in the optimization process. By combining RSM, Fuzzy AHP, and Fuzzy CoCoSo, this study not only optimizes parameters but also integrates the complexity and uncertainty inherent in machining magnesium alloys. In conclusion, this work provides a more robust and adaptable optimization solution than conventional methods and other hybrid approaches. The integration of fuzzy logic with RSM allows for a more flexible and comprehensive decision-making process, ensuring improved machining performance, sustainability, and cost-effectiveness when applied to the machining of magnesium alloys. The results offer valuable insights into how this hybrid framework can enhance industrial applications and contribute to more efficient and sustainable manufacturing practices.

## Materials and methods

**Fig. 1:**
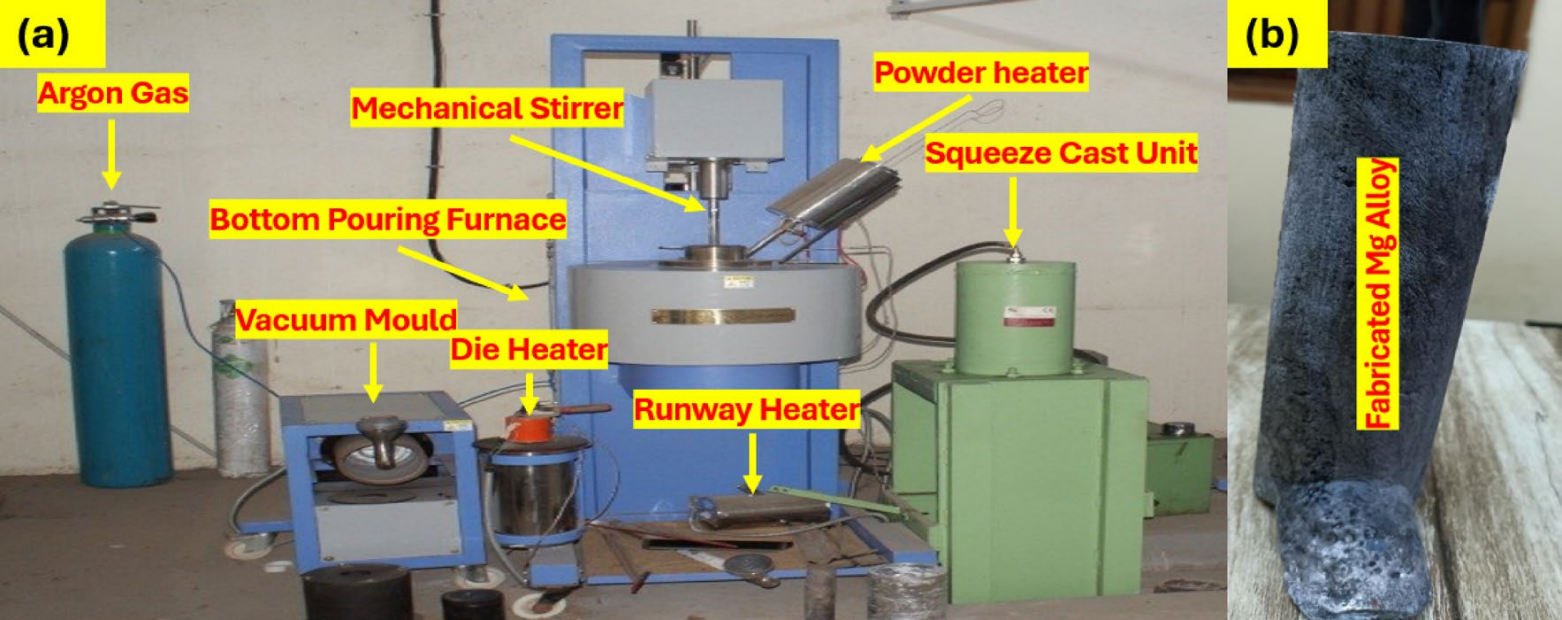
(**a**) Experimental Setup, (**b**) Casted Mg Sample.

In this study, commercial pure magnesium (Mg) with a purity of 99.9% and silicon (Si), calcium (Ca), and zinc (Zn) as alloying elements were produced by the bottom pouring stir casting technique. Mg, Si (1.22%), Ca (0.54), and Zn (0.54) were purchased from Ms Vaishnavi Metals, Chennai, with 99.8% purity. Figure 1 shows that the stir-casting machine fabricated the samples with bottom pouring. The furnace used to melt this Mg-Si-(Ca-Zn) alloy was a Bottom pouring resistance furnace at SRM KTR Foundry lab with a temperature of 950 °C. Figure 1 shows that the stir-casting machine fabricated the samples with bottom pouring. The casting process took 2 h; the melting temperature was between 850 °C and 900 °C. First, the crucible in the furnace was pre-heated at a temperature between 850 °C and 950 °C for 45 min. Later, the base metal Magnesium was added and melted for 45 min in the crucible, after which the remaining Silicon, Calcium, and Zinc metals were added in the crucible of the furnace concerning the alloy composition, and the melt was protected from oxidation by N2 + 0.1% SF6. Then, the melt was cast into a permanent wedge mould, which was cylindrical. Later, the mould kept cooling for more than 2 h.

**Table 1:** Process Parameter.

Factor	Name	Units	Min	Max	Response
A	Cutting Speed	m/min	11.00	42.00	(1) Surface Roughness (µm), (2) MMR (cm3/min)
B	Feed rate	mm/rev	0.0500	0.150	3. Energy Consumption (w), 4. Tool Wear (µ)
C	Percentage intensity of ultrasonic power	%	0.0000	100.00	5. Dimensional Accuracy (%)

The ranges of process parameters—cutting speed (11.00 to 42.00 m/min), feed rate (0.0500 to 0.150 mm/rev), and ultrasonic power intensity (0.0000% to 100.00%)—have been clearly mentioned in Table 1. These ranges were selected based on prior research and the capabilities of the machining equipment, ensuring their relevance to the industrial machining of magnesium alloys. By varying these parameters within the specified ranges, the study aims to optimize the machining process for improved Surface Roughness (µm),, Tool Wear (µ), material removal rate (MMR), energy consumption (w), and dimensional accuracy (%), all of which are critical for efficient and sustainable manufacturing.

### Experimental setup

**Fig. 2:**
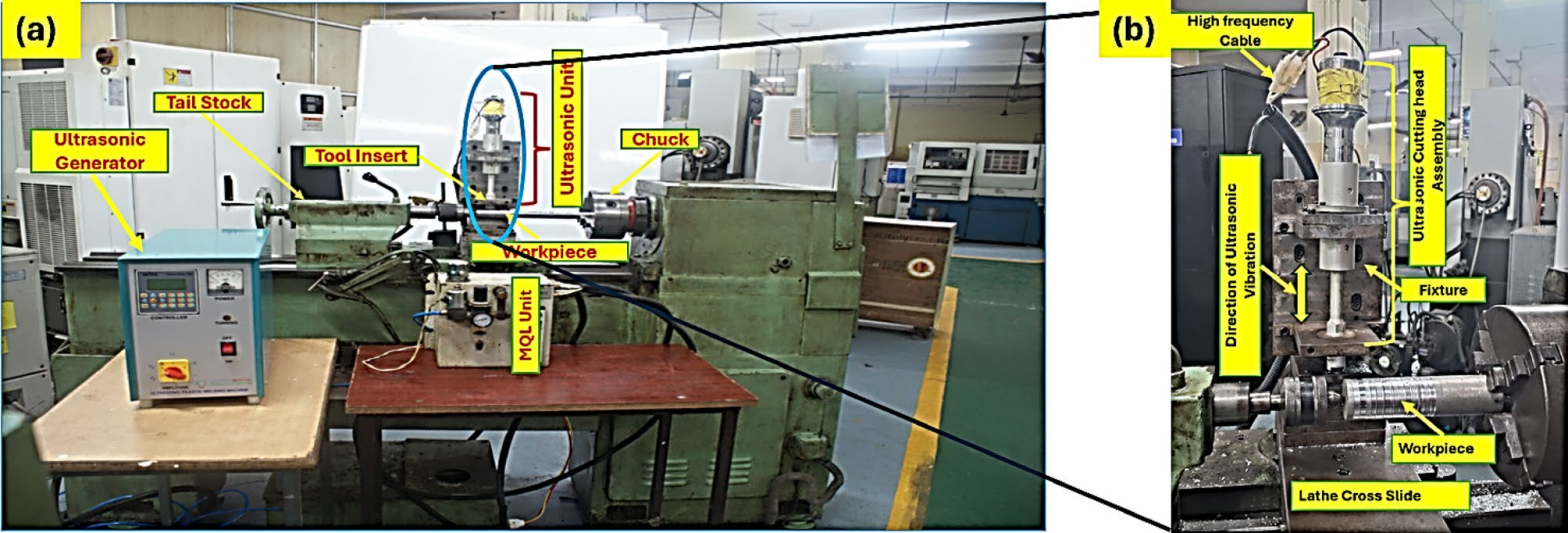
(**a**) Machining Set up, (**b**) Set up of Ultrasonic Assisted Turning.

Figure 2(a) illustrates the machining setup, while Fig. 2(b) provides a detailed depiction of the ultrasonic-assisted turning (UAT) experimental setup employed in this study. The setup includes an ultrasonic-assisted turning head and an ultrasonic generator. The UAT of the Mg-SiC alloy was conducted using a PSG A141 center lathe with a power capacity of 2.24 kW and a spindle speed range of 38–1600 rpm.

The UAT head consists of an ultrasonic transducer, a booster, and a horn. The ultrasonic generator increases the frequency of the electrical energy to 20 kHz, which is then supplied to the ultrasonic transducer, converting high-frequency electrical energy into mechanical vibrations. The booster amplifies the amplitude of these mechanical vibrations, while the stepped horn reduces the vibration amplitude and simultaneously transfers the vibration energy to the cutting tool. The tool insert is securely attached to the horn’s 20 mm diameter end using a screw. Both the horn and booster are made from aluminum alloy. The horn has a calculated length of 130 mm, with smaller and larger end diameters of 16 mm and 20 mm, respectively. The resulting vibration parameters include a frequency of 20 kHz and an amplitude of 10 μm. The UAT head is mounted on the lathe using a specially designed fixture that ensures the tool insert vibrates tangentially to the rotating workpiece. This setup facilitates the longitudinal vibration mode of the UAT head, allowing precise control over the vibrations and enabling accurate, efficient machining.

### General framework of the execution system

The general framework of the execution system integrates a hybrid optimization approach combining Response Surface Methodology (RSM) and Fuzzy Combined Compromise Solution (CoCoSo) to optimize the machining parameters in ultrasonic-assisted turning (UAT) for magnesium alloys.

**Fig. 3:**
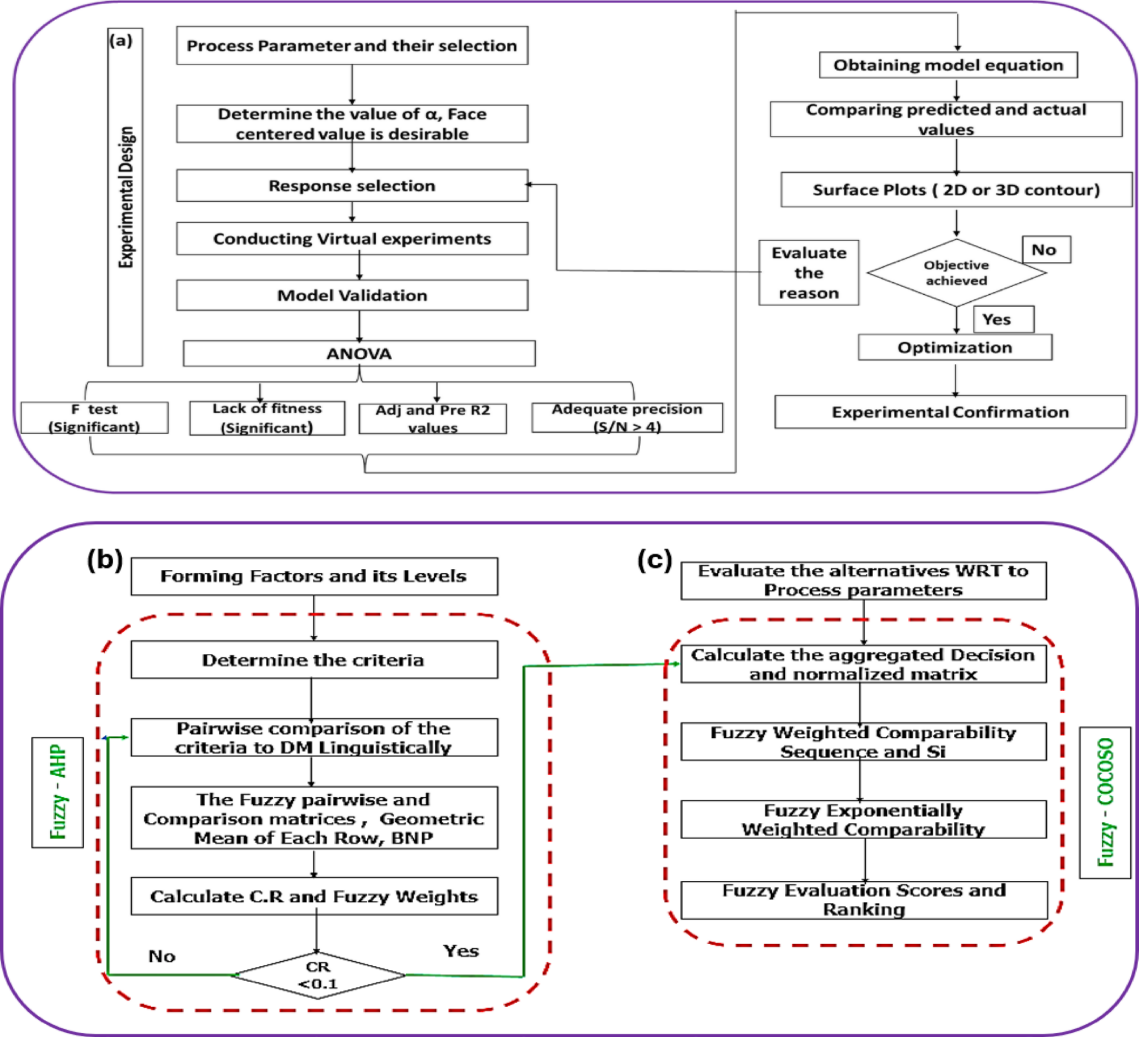
General Framework of the execution system, (**a**) RSM- The Experimental Design, (**b**) Fuzzy AHP- Determination of weightage, (**c**) Fuzzy COCOSO- Determination of Ranking Alternatives.

#### Experimental design

The first phase involves experimental design using RSM, specifically the Hybrid Box-Behnken model, to systematically select and optimize the experimental parameters in Fig. 3(a). This approach allows for an efficient exploration of the effect of cutting speed, feed rate, and ultrasonic power intensity on machining performance^73–80^. The RSM design includes conducting virtual experiments and model validation through ANOVA, where the F-test is used to assess parameter significance. A total of 15 experimental runs were carried out in a quadratic design to evaluate surface roughness, tool wear, material removal rate (MMR), energy consumption, and dimensional accuracy. The model’s predictions were then validated through confirmation experiments to ensure reliability.

#### Fuzzy AHP

Following the experimental design, Fuzzy AHP is employed solely for determining the fuzzy criteria weights for the various process parameters, as shown in Fig. 3(b). In this phase, the importance of each parameter—such as cutting speed, feed rate, and ultrasonic power intensity—is evaluated through pairwise comparisons conducted linguistically by decision makers. The fuzzy pairwise comparison matrices are used to calculate the geometric mean of each row, and the consistency ratio (CR) is checked to ensure it is below the threshold of 0.1, confirming consistency in the judgments. All calculations are carried out using MS Excel, ensuring transparency and accuracy.

Fuzzy AHP works in conjunction with RSM by providing a clear and structured method for determining the relative importance of each process parameter after the RSM experiment has been designed. While RSM focuses on selecting and optimizing the experimental parameters based on their effects on machining performance, Fuzzy AHP assigns relative weights to these parameters. The results from RSM (e.g., the significance of cutting speed, feed rate, and ultrasonic power intensity) are further refined through Fuzzy AHP, which quantifies the importance of each factor in a decision-making framework. In this way, Fuzzy AHP does not alter the experimental design but enhances the optimization process by incorporating expert judgment into the decision-making criteria. This allows for a more comprehensive approach, where RSM identifies the optimal process conditions and Fuzzy AHP fine-tunes the focus on the most important parameters. Together, they provide a robust framework for optimizing the UAT process for magnesium alloys.

However, while the article explains the experimental setup, a more detailed description of the computational procedure, such as the steps involved in calculating fuzzy weights and decision matrices, is required for a deeper understanding of the computational process. These steps include constructing fuzzy pairwise comparison matrices, applying the fuzzy synthetic extent method, and calculating the fuzzy weights for each parameter. The decision matrices, once established, are used to evaluate and rank the alternatives based on the fuzzy weights, ultimately selecting the optimal machining parameters for UAT. These detailed steps ensure that subjective judgments are effectively integrated into the decision-making process, improving the robustness and reliability of the optimization model.

#### Fuzzy CoCoSo

The final phase utilizes Fuzzy CoCoSo to determine the optimal process parameters by evaluating the alternatives relative to the chosen criteria, as illustrated in Fig. 3(c). This method calculates an aggregated decision matrix that incorporates the fuzzy weighted comparability sequences derived from Fuzzy AHP to rank the alternatives. Fuzzy Exponentially Weighted Comparability is then used to refine these rankings, and the final evaluation scores are employed to select the best-performing parameter set. The combination of RSM, Fuzzy AHP, and Fuzzy CoCoSo forms a synergistic framework for parameter optimization. RSM is used in the initial phase to design experiments and analyze the effects of various process parameters (cutting speed, feed rate, and ultrasonic power intensity) on the machining outcomes. After identifying the most significant parameters, Fuzzy AHP comes into play to assign relative importance (weights) to these parameters based on expert judgment, ensuring a subjective yet systematic approach to prioritization.

In the final phase, Fuzzy CoCoSo takes the weighted parameters from Fuzzy AHP and evaluates different alternatives or process configurations relative to these weighted criteria. By using fuzzy logic, Fuzzy CoCoSo accommodates the inherent uncertainty in the decision-making process and provides a robust ranking of alternatives. This allows the system to not only identify the optimal combination of parameters but also account for multiple conflicting criteria, such as surface quality, tool wear, material removal rate (MMR), and energy consumption. Thus, while RSM identifies the experimental setup and evaluates the direct impact of process parameters, Fuzzy AHP prioritizes these parameters, and Fuzzy CoCoSo ranks alternatives based on their weighted importance. This integrated approach offers a comprehensive solution for parameter optimization, ensuring both precision in machining and sustainability in manufacturing.

## Results and discussion

**Table 2:** Experimental Response.

Run	Factor 1	Factor 2	Factor 3	Response 1	Response 2	Response 3	Response 4	Response 5
	A: Cutting Speed	B: Feed rate	C: Intensity of ultrasonic power	Surface Roughness	MMR	Energy Consumption	Tool Wear	Dimensional Accuracy
	m/min	mm/rev	%	µm	cm3/min	w	µ	%
1	26.5	0.1	100	0.68	0.9	150	55	98
2	42	0.1	17	0.7	1	170	65	95
3	26.5	0.1	17	0.85	0.8	140	50	96
4	11	0.1	100	1.1	0.6	120	45	92
5	42	0.05	0	0.95	0.75	190	80	90
6	26.5	0.15	100	0.72	0.95	160	60	97
7	26.5	0.05	17	0.9	0.7	135	58	94
8	42	0.1	100	0.65	1.05	180	70	93
9	26.5	0.05	100	0.6	0.85	140	52	96
10	42	0.15	0	1.05	1.15	200	85	89
11	26.5	0.15	17	0.88	0.85	155	54	94
12	11	0.05	0	1.3	0.55	110	40	91
13	26.5	0.1	0	1.15	0.7	145	65	90
14	11	0.1	17	1.12	0.6	125	48	93
15	11	0.15	0	1.25	0.65	130	50	91

The experimental results from Table 2 illustrate the effects of varying cutting speed (m/min), feed rate (mm/rev), and ultrasonic power intensity (%) on surface roughness (µm), material removal rate (MMR, cm³/min), energy consumption (w), tool wear (µ), and dimensional accuracy (%). A total of 15 runs were conducted, such as in Run 1 where a cutting speed of 26.5 m/min, feed rate of 0.1 mm/rev, and ultrasonic power intensity of 100% yielded a surface roughness of 0.68 μm, MMR of 0.9 cm³/min, energy consumption of 150 w, tool wear of 55 µ, and dimensional accuracy of 98%. The responses varied across different runs, with lower cutting speeds generally leading to higher surface roughness and tool wear. This study highlights the significance of machining parameters on key performance responses.

**Table 3:** Membership function of linguistic Scale.

Fuzzy Number	9	8	7	6	5	4	3	2	1
Linguistic	Perfect -P	Absolute- A	Very Good -VG	Fairly Good - FG	Good -G	Preferable - P	Not Bad - NB	Weak Advantage - WA	Equal -E
The scale of the Fuzzy number	(8.9,10)	(7,8,9)	(6,7,8)	(5,6,7)	(4,5,6)	(3,4,5)	(2,3,4)	(1,2,3)	(1,1,1)

In Table 3, each linguistic term corresponds to a fuzzy number and a scale. For example, the linguistic term “Perfect” (P) is represented by the fuzzy number 9 with a scale of (8.9, 10), “Absolute” (A) corresponds to 8 with a scale of (7, 8, 9), and so on, down to “Equal” (E) with a fuzzy number of 1 and a scale of (1, 1, 1). This structure is essential in the Fuzzy AHP method for transforming subjective judgments into quantifiable fuzzy numbers, allowing for more nuanced decision-making.

### Initial comparison matrices

**Table 4:** Initial comparison Matrices.

Alt	Left Criteria Is Greater										Right Criteria Is Greater									Number of Experts
		P	A	VG	FG	G	P	NB	W	Equal	WA	NB	P	G	FG	VG	A	P		
A	-	-	-	-	-	-	-	3	4	2	2	1	-	-	-	-	-	-	B	12
A	-	-	-	-	-	-	-	2	2	4	3	1	-	-	-	-	-	-	C	12
A	-	-	-	-	-	-	-	1	2	3	4	2	-	-	-	-	-	-	D	12
A	-	-	-	-	-	-	-	3	3	3	2	1	-	-	-	-	-	-	E	12
A	-	-	-	-	-	-	-	4	3	3	2		-	-	-	-	-	-	F	12
A	-	-	-	-	-	-	-	3	4	3	2		-	-	-	-	-	-	G	12
A	-	-	-	-	-	-	-	3	3	3	2	1	-	-	-	-	-	-	H	12
A	-	-	-	-	-	-	-		3	3	3	2	1	-	-	-	-	-	I	12
A	-	-	-	-	-	-	-	2	4	3	2	1		-	-	-	-	-	J	12
A	-	-	-	-	-	-	-		3	3	3	2	1	-	-	-	-	-	K	12
A	-	-	-	-	-	-	-	3	3	3	2	1	-	-	-	-	-	-	L	12
A	-	-	-	-	-	-	-	3	3	3	2	1	-	-	-	-	-	-	M	12
A	-	-	-	-	-	-	-	2	4	3	2	1	-	-	-	-	-	-	N	12
A	-	-	-	-	-	-	-		3	3	3	2	1	-	-	-	-	-	-	-
-	-	-	-	-	-	-	-	-	-	-	-	-	-	-	-	-	-	-	-	-
-	-	-	-	-	-	-	-	-	-	-	-	-	-	-	-	-	-	-	-	-
K	-	-	-	-	-	-	-	-		3	3	3	2	1	-	-	-	-	L	12
K	-	-	-	-	-	-	-	-	3	3	3	2	1	-	-	-	-	-	M	12
K	-	-	-	-	-	-	-	3	3	3	2	1		-	-	-	-	-	N	12
K	-	-	-	-	-	-	-	3	3	3	2	1		-	-	-	-	-	O	12
L	-	-	-	-	-	-	1	2	3	3	2	1		-	-	-	-	-	M	12
L	-	-	-	-	-		3	3	2	3	1	-		-	-	-	-	-	N	12
L	-	-	-	-	-	3	3	3	2	1	-	-		-	-	-	-	-	O	12
M	-	-	-	-	-	-	-	-	-	-	-	-	-	-	-	-	-	-	N	12
M	-	-	-	-	-	-	-	-	-	-	-	-	-	-	-	-	-	-	O	12
N	-	-	-	-	-	-	-		3	3	3	2	1	-	-	-	-	-	O	12

Table 4 outlines the Initial Comparison Matrices for the Fuzzy AHP approach, comparing multiple criteria based on expert input. The table presents comparisons where one criterion is deemed greater than another by various experts. The criteria are presented in pairs under the headings “Left Criteria Is Greater” and “Right Criteria Is Greater,” with each pair assigned a linguistic rating such as Perfect (P), Absolute (A), Very Good (VG), Fairly Good (FG), Good (G), Preferable (P), Not Bad (NB), Weak Advantage (WA), or Equal (E) to indicate the relative preference. Each comparison set involves 12 experts, and their assessments are distributed across the linguistic scale. For instance, when comparing criteria A and B, experts rated them across a range of “Not Bad,” “Weak Advantage,” and “Equal.” At the same time, other comparisons follow similar patterns across different criteria pairs. This comparison process is foundational in establishing the relative importance of criteria for further fuzzy-based analysis.

### Integrated fuzzy comparison matrix

**Table 5:** Integrated fuzzy comparison Matrix.

	A			B			C			-	M			*N*			O		
A	1.00	1.00	1.00	0.88	1.35	1.93	0.76	1.03	1.43	-	0.88	1.27	1.76	0.83	1.23	1.72	0.53	0.74	1.07
B	0.52	0.74	1.13	1.00	1.00	1.00	0.88	1.27	1.76	-	0.53	0.74	1.07	0.88	1.27	1.76	0.83	1.23	1.72
C	0.70	0.97	1.32	0.57	0.79	1.13	1.00	1.00	1.00	-	0.53	0.74	1.07	0.53	0.74	1.07	0.88	1.27	1.76
D	0.83	1.23	1.72	0.58	0.81	1.20	0.43	0.56	0.82	-	0.88	1.27	1.76	0.88	1.27	1.76	1.43	2.09	2.78
E	0.57	0.79	1.13	0.57	0.79	1.13	0.57	0.79	1.13	-	1.43	2.09	2.78	1.43	2.09	2.78	0.88	1.27	1.76
F	0.48	0.65	0.95	0.48	0.65	0.95	0.48	0.65	0.95	-	0.88	1.13	1.46	1.43	2.09	2.78	0.88	1.27	1.76
G	0.49	0.68	1.01	0.55	0.79	1.17	0.48	0.65	0.95	-	0.88	1.27	1.76	0.53	0.74	1.07	0.53	0.74	1.07
H	0.57	0.79	1.13	0.57	0.79	1.13	0.93	1.35	1.90	-	1.43	2.09	2.78	0.88	1.27	1.76	0.51	0.72	1.01
I	0.93	1.35	1.90	1.01	1.35	1.72	0.28	0.35	0.46	-	1.43	2.09	2.78	1.43	2.09	2.78	0.88	1.27	1.76
J	0.58	0.81	1.20	0.93	1.35	1.90	0.36	0.48	0.70	-	0.88	1.27	1.76	0.81	1.20	1.76	0.53	0.74	1.07
K	0.93	1.35	1.90	0.49	0.68	1.01	0.25	0.32	0.45	-	0.53	0.74	1.07	0.88	1.27	1.76	0.88	1.27	1.76
L	0.57	0.79	1.13	0.93	1.35	1.90	0.93	1.35	1.90	-	0.91	1.30	1.79	1.43	1.97	2.54	2.21	3.12	3.97
M	0.57	0.79	1.13	0.93	1.35	1.90	0.93	1.35	1.90	-	1.00	1.00	1.00	0.53	0.74	1.07	0.83	1.23	1.72
N	0.58	0.81	1.20	0.57	0.79	1.13	0.93	1.35	1.90	-	0.93	1.35	1.90	1.00	1.00	1.00	0.53	0.74	1.07
O	0.93	1.35	1.90	0.58	0.81	1.20	0.57	0.79	1.13	-	0.58	0.81	1.20	0.93	1.35	1.90	1.00	1.00	1.00

Table 5 shows the Integrated Fuzzy Comparison Matrix used in the Fuzzy AHP method. This matrix provides pairwise comparisons between criteria (A, B, C, D, E, etc.) in fuzzy number form. The comparisons are expressed as fuzzy numbers, with values representing the minimum, mean, and maximum boundaries of the fuzzy set for each criterion comparison. For example, the comparison between criterion A and B is defined as (0.52, 0.74, 1.13), indicating a range where A is weaker than B. Similarly, other comparisons are provided, such as A vs. C with fuzzy numbers (0.70, 0.97, 1.32), and D vs. M with (0.88, 1.27, 1.76). The diagonal elements of the matrix have a value of 1 (e.g., A vs. A) as criteria are equally important when compared to themselves. The matrix helps quantify expert judgments, turning qualitative assessments into measurable data for ranking criteria using fuzzy logic.

**Table 6:** Fuzzy geometric mean and Weights.

Alternatives	Fuzzy Geometric Mean of Each Row			Fuzzy Weights			BNP	Normalization
1st	0.782	1.099	1.510	0.037	0.072	0.136	0.082	0.072
2nd	0.761	1.065	1.462	0.036	0.070	0.132	0.079	0.070
3rd	0.904	1.252	1.682	0.043	0.082	0.152	0.092	0.081
4th	0.815	1.131	1.538	0.039	0.074	0.139	0.084	0.074
5th	1.022	1.410	1.889	0.049	0.092	0.170	0.104	0.091
6th	0.663	0.893	1.222	0.032	0.059	0.110	0.067	0.059
7th	0.627	0.856	1.192	0.030	0.056	0.107	0.065	0.057
8th	0.721	0.987	1.335	0.035	0.065	0.120	0.073	0.064
9th	0.793	1.079	1.434	0.038	0.071	0.129	0.079	0.070
10th	0.659	0.907	1.268	0.032	0.059	0.114	0.068	0.060
11th	0.585	0.789	1.100	0.028	0.052	0.099	0.060	0.052
12th	0.929	1.285	1.743	0.045	0.084	0.157	0.095	0.084
13th	0.614	0.832	1.156	0.029	0.055	0.104	0.063	0.055
14th	0.579	0.784	1.102	0.028	0.051	0.099	0.060	0.052
15th	0.645	0.877	1.227	0.031	0.058	0.111	0.066	0.058

Table 6 presents the Fuzzy Geometric Mean and Weights used to calculate the relative importance of each criterion in a Fuzzy AHP analysis. Each row’s fuzzy geometric mean (ranging from minimum to maximum values) and corresponding fuzzy weights are displayed. The fuzzy weights are calculated by dividing the fuzzy geometric mean by the total sum of all geometric means to normalize the values. For example, the first row has a fuzzy geometric mean of (0.782, 1.099, 1.510) and corresponding fuzzy weights of (0.037, 0.072, 0.136), while the normalized value is 0.072. This process transforms subjective judgments into weighted values that contribute to decision-making, with the higher weights indicating more significant criteria. The normalized weights reflect the proportionate influence of each criterion in the decision-making model.

**Table 7:** Linguistic Variables.

Linguistic Variables	Very High	High	Medium High	Medium	Medium Low	Low	Very Low
Fuzzy numbers	(0.9,1.0,1.0)	(0.7,0.9,1.0)	(0.5,0.7,0.9)	(0.3,0.5,0.7)	(0.1,0.3.0.5)	(0.0,0.1,0.3)	(0.0,0.0,0.1)

Table 7 defines the Linguistic Variables used in the fuzzy logic system, which are mapped to corresponding fuzzy numbers. The variables range from Very High to Very Low, each represented by a triangular fuzzy number. For example, “Very High” is associated with the fuzzy number (0.9, 1.0, 1.0), indicating a strong belief that the value is close to 1. “High” is (0.7, 0.9, 1.0), “Medium High” is (0.5, 0.7, 0.9), and so on, down to “Very Low” with a fuzzy number of (0.0, 0.0, 0.1). These fuzzy numbers help quantify qualitative judgments and are crucial for calculations in Fuzzy AHP and Fuzzy Logic Decision-Making models.

**Table 8:** Criteria for decision Matrix.

Alternatives	1 st criteria							2nd criteria							--	14th criteria							15th criteria						
	VL	L	ML	M	MH	H	VH	VL	L	ML	M	MH	H	VH	-	VL	L	ML	M	MH	H	VH	VL	L	ML	M	MH	H	VH
1st	-	-	-		2	2	4			1	2	1	3	1	-		4	2	2					2	4	2			
2nd	-	-	-	2	2	4	-			2	4	2		-	-	4	1	3					1	3	1	3			
3rd	-	-	2	2	3	1	-			2	3	2	1	-	-	1	1	4	2					2	2	4			
4th	-	-	-	3	4	1	-					4	4	-	-	2	5	1							5	1			
5th	1	4	-	1	-		-	4	3	1			-	-	-	2	3	2	1				1	2	3	2			
6th	-		-	3	-		-	2	1	3	1		-	-	-					4	2	2					2	4	2
7th	-	2	-	2	-		-	4	2				-	-	-				4	1	3					1	3	1	3
8th	-	2	-	1	-		-	3	2	1			-	-	-				1	1	4	2					2	2	4
9th	-	3	-	1	-		-		4	4			-	-	-				2	5	1							5	1
10th	-	1	-		-		-		1		3		-	-	-				2	3	2	1				1	2	3	2
11th	-	-	-	2	-	4	-			3	4	1	-	-	-			4	4		-	-			2	4	2		
12th	-	-	-	4	-		-			2	1	3	1	-	-		3	3	2		-	-		1	3	1	3		
13th	-	-	-	3	-		-			4	2			-	-			2	2	4	-	-			2	2	4		
14th	-	-	-	4	-		-			3	2	1		-	-		1	2	3	2	-	-				5	1		
15th	-	-	-		-		-				4	4		-	-		2	4		1	-	-		1	2	3	2		

Table 8 presents the Criteria Ratings for Alternatives across 15 criteria, with linguistic values ranging from Very Low (VL) to Very High (VH). Each row corresponds to an alternative, and the columns represent ratings for the alternatives based on the 1 st to 15th criteria. For instance, the 1 st alternative is rated as Medium High (MH) for the 4th criterion, Medium (M) for the 6th criterion, and Low (L) for the 9th criterion. Each alternative is assessed across a diverse set of criteria using linguistic variables, reflecting the qualitative judgments of each criterion’s importance for that alternative.

### Aggregated decision matrix

**Table 9:** Aggregated decision Matrix.

Alternatives	0.04	0.07	0.14	0.04	0.07	0.13	0.04	0.08	0.15	-	0.03	0.05	0.10	0.03	0.05	0.10	0.03	0.06	0.11
	X1			X2			X3			-	X13			X14			X15		
A1	0.40	0.48	0.52	0.28	0.38	0.45	0.20	0.31	0.41	-	0.03	0.11	0.21	0.05	0.13	0.24	0.07	0.16	0.27
A2	0.29	0.40	0.48	0.16	0.27	0.37	0.20	0.31	0.41	-	0.01	0.06	0.15	0.02	0.07	0.15	0.07	0.14	0.24
A3	0.20	0.31	0.41	0.19	0.29	0.39	0.24	0.35	0.45	-	0.09	0.19	0.29	0.07	0.15	0.25	0.09	0.19	0.29
A4	0.24	0.35	0.45	0.32	0.43	0.51	0.27	0.37	0.46	-	0.06	0.14	0.24	0.01	0.05	0.15	0.05	0.13	0.21
A5	0.03	0.10	0.20	0.01	0.04	0.12	0.21	0.32	0.42	-	0.02	0.06	0.14	0.03	0.09	0.19	0.06	0.14	0.24
A6	0.17	0.28	0.39	0.04	0.10	0.18	0.04	0.11	0.20	-	0.32	0.43	0.51	0.35	0.44	0.51	0.37	0.47	0.52
A7	0.07	0.16	0.27	0.00	0.01	0.07	0.03	0.10	0.20	-	0.25	0.36	0.45	0.25	0.36	0.45	0.35	0.43	0.49
A8	0.04	0.11	0.19	0.01	0.03	0.09	0.05	0.14	0.24	-	0.40	0.48	0.52	0.36	0.45	0.51	0.40	0.48	0.52
A9	0.05	0.13	0.24	0.03	0.11	0.21	0.07	0.16	0.27	-	0.35	0.44	0.50	0.25	0.36	0.46	0.29	0.37	0.40
A10	0.00	0.01	0.02	0.06	0.11	0.16	0.04	0.11	0.21	-	0.23	0.32	0.40	0.29	0.39	0.47	0.35	0.44	0.50
A11	0.29	0.40	0.48	0.13	0.24	0.35	0.31	0.41	0.48	-	0.11	0.21	0.32	0.11	0.21	0.32	0.16	0.27	0.37
A12	0.16	0.27	0.37	0.18	0.27	0.36	0.31	0.41	0.49	-	0.06	0.15	0.25	0.06	0.15	0.25	0.14	0.24	0.35
A13	0.11	0.19	0.27	0.07	0.15	0.23	0.35	0.45	0.51	-	0.19	0.29	0.40	0.19	0.29	0.40	0.19	0.29	0.40
A14	0.13	0.24	0.35	0.09	0.17	0.25	0.37	0.46	0.51	-	0.14	0.24	0.35	0.14	0.24	0.35	0.13	0.21	0.29
A15	0.01	0.02	0.03	0.21	0.32	0.43	0.32	0.42	0.49	-	0.06	0.14	0.23	0.06	0.14	0.23	0.14	0.24	0.35
MAX	0.40	0.48	0.52	0.32	0.43	0.51	0.37	0.46	0.51	-	0.40	0.48	0.52	0.36	0.45	0.51	0.40	0.48	0.52
MIN	0.00	0.01	0.02	0.00	0.01	0.07	0.03	0.10	0.20	-	0.01	0.06	0.14	0.01	0.05	0.15	0.05	0.13	0.21
MAX-MIN	0.52	0.52	0.52	0.51	0.51	0.51	0.49	0.49	0.49	-	0.51	0.51	0.51	0.50	0.50	0.50	0.47	0.47	0.47

Table 9 presents the aggregated decision matrix for 15 alternatives (A1 to A15) across multiple criteria, with each alternative assigned fuzzy values representing lower, middle, and upper bounds. For instance, A1 has (0.40, 0.48, 0.52) values for the first criterion, while A6 shows higher values of (0.32, 0.43, 0.51) values for the sixth criterion. The table also includes the highest (MAX), lowest (MIN), and the range (MAX-MIN) of values for each criterion across all alternatives. As shown in Table 9, this matrix supports decision-making methods like fuzzy CoCoSo by highlighting variability and uncertainty in the criteria evaluations. The MAX-MIN range emphasises differences in the evaluations.

### Normalized matrix

**Table 10:** Normalized Matrix.

Alternatives	0.04	0.07	0.14	0.04	0.07	0.13	0.04	0.08	0.15	-	0.03	0.05	0.10	0.03	0.05	0.10	0.03	0.06	0.11
	X1			X2			X3				X13			X14			X15		
A1	0.77	0.92	1.00	0.55	0.75	0.89	0.22	0.42	0.64	-	0.03	0.18	0.39	0.09	0.25	0.47	0.03	0.23	0.46
A2	0.56	0.77	0.92	0.32	0.53	0.74	0.21	0.42	0.64	-	0.00	0.09	0.26	0.03	0.12	0.28	0.03	0.19	0.40
A3	0.38	0.59	0.78	0.37	0.58	0.78	0.14	0.34	0.56	-	0.16	0.34	0.55	0.12	0.29	0.49	0.09	0.29	0.51
A4	0.46	0.67	0.86	0.63	0.84	1.00	0.11	0.29	0.51	-	0.09	0.25	0.45	0.00	0.09	0.28	0.00	0.17	0.34
A5	0.06	0.19	0.38	0.01	0.08	0.24	0.19	0.40	0.62	-	0.01	0.09	0.25	0.05	0.17	0.36	0.01	0.19	0.40
A6	0.33	0.54	0.74	0.08	0.20	0.36	0.64	0.84	0.97	-	0.61	0.82	0.97	0.68	0.87	1.00	0.69	0.89	1.00
A7	0.13	0.31	0.51	0.00	0.03	0.13	0.64	0.85	1.00	-	0.47	0.68	0.87	0.49	0.71	0.88	0.63	0.81	0.94
A8	0.08	0.21	0.36	0.01	0.07	0.18	0.56	0.77	0.95	-	0.76	0.92	1.00	0.71	0.89	1.00	0.74	0.91	1.00
A9	0.09	0.26	0.46	0.05	0.21	0.42	0.51	0.73	0.90	-	0.66	0.84	0.96	0.49	0.71	0.91	0.51	0.67	0.74
A10	0.00	0.01	0.04	0.12	0.21	0.32	0.62	0.82	0.97	-	0.43	0.61	0.76	0.57	0.77	0.93	0.63	0.83	0.96
A11	0.56	0.77	0.92	0.26	0.47	0.68	0.07	0.22	0.42	-	0.18	0.39	0.61	0.20	0.41	0.63	0.23	0.46	0.69
A12	0.31	0.51	0.72	0.36	0.54	0.71	0.04	0.21	0.42	-	0.09	0.26	0.47	0.11	0.28	0.49	0.19	0.40	0.63
A13	0.21	0.36	0.51	0.13	0.29	0.45	0.01	0.14	0.34	-	0.34	0.55	0.76	0.36	0.57	0.79	0.29	0.51	0.74
A14	0.26	0.46	0.67	0.18	0.34	0.50	0.00	0.11	0.29	-	0.25	0.45	0.66	0.27	0.47	0.68	0.17	0.34	0.51
A15	0.01	0.04	0.06	0.42	0.63	0.84	0.04	0.19	0.40	-	0.09	0.25	0.43	0.11	0.27	0.45	0.19	0.40	0.63

Table 10 presents the normalized matrix for 15 alternatives (A1 to A15) across different criteria (X1 to X15), with the values standardized to a common scale. Each alternative is assigned normalized values, for instance, A1 exhibits values like (0.77, 0.92, 1.00) for X1, X2, and X3, respectively. Similarly, A6 shows values like (0.33, 0.54, 0.74) for the same criteria, but its highest normalized values (0.69, 0.89, 1.00) appear for X14 and X15. The normalized matrix helps compare alternatives based on their performance across all criteria by eliminating scale differences.

### Fuzzy weighted comparability sequence and S_i_

**Table 11:** Fuzzy weighted comparability sequence and Si.

Alt	X1			X2			X3			-	X13			X14			X15		
A1	0.03	0.07	0.14	0.02	0.05	0.12	0.01	0.03	0.10	-	0.00	0.01	0.04	0.00	0.01	0.05	0.00	0.01	0.05
A2	0.02	0.06	0.13	0.01	0.04	0.10	0.01	0.03	0.10	-	0.00	0.01	0.03	0.00	0.01	0.03	0.00	0.01	0.04
A3	0.01	0.04	0.11	0.01	0.04	0.10	0.01	0.03	0.09	-	0.00	0.02	0.06	0.00	0.02	0.05	0.00	0.02	0.06
A4	0.02	0.05	0.12	0.02	0.06	0.13	0.00	0.02	0.08	-	0.00	0.01	0.05	0.00	0.00	0.03	0.00	0.01	0.04
A5	0.00	0.01	0.05	0.00	0.01	0.03	0.01	0.03	0.09	-	0.00	0.01	0.03	0.00	0.01	0.04	0.00	0.01	0.04
A6	0.01	0.04	0.10	0.00	0.01	0.05	0.03	0.07	0.15	-	0.02	0.04	0.10	0.02	0.04	0.10	0.02	0.05	0.11
A7	0.00	0.02	0.07	0.00	0.00	0.02	0.03	0.07	0.15	-	0.01	0.04	0.09	0.01	0.04	0.09	0.02	0.05	0.10
A8	0.00	0.01	0.05	0.00	0.00	0.02	0.02	0.06	0.14	-	0.02	0.05	0.10	0.02	0.05	0.10	0.02	0.05	0.11
A9	0.00	0.02	0.06	0.00	0.01	0.06	0.02	0.06	0.14	-	0.02	0.05	0.10	0.01	0.04	0.09	0.02	0.04	0.08
A10	0.00	0.00	0.01	0.00	0.01	0.04	0.03	0.07	0.15	-	0.01	0.03	0.08	0.02	0.04	0.09	0.02	0.05	0.11
A11	0.02	0.06	0.13	0.01	0.03	0.09	0.00	0.02	0.06	-	0.01	0.02	0.06	0.01	0.02	0.06	0.01	0.03	0.08
A12	0.01	0.04	0.10	0.01	0.04	0.09	0.00	0.02	0.06	-	0.00	0.01	0.05	0.00	0.01	0.05	0.01	0.02	0.07
A13	0.01	0.03	0.07	0.00	0.02	0.06	0.00	0.01	0.05	-	0.01	0.03	0.08	0.01	0.03	0.08	0.01	0.03	0.08
A14	0.01	0.03	0.09	0.01	0.02	0.07	0.00	0.01	0.04	-	0.01	0.02	0.07	0.01	0.02	0.07	0.01	0.02	0.06
A15	0.00	0.00	0.01	0.02	0.04	0.11	0.00	0.02	0.06	-	0.00	0.01	0.05	0.00	0.01	0.05	0.01	0.02	0.07

Table 11 displays the Fuzzy Weighted Comparability Sequence and Si for 15 alternatives (A1 to A15) across several criteria (X1 to X15). The fuzzy weighted comparability values range from low to high, with each alternative evaluated on its relative importance across different factors. For instance, A1 has fuzzy values such as (0.03, 0.07, 0.14) for X1, X2, and X3, respectively, while A6 has values like (0.01, 0.04, 0.10) for the same criteria. Additionally, A6 shows significant X13, X14, and X15 values like (0.02, 0.04, 0.10) and (0.02, 0.05, 0.11), indicating stronger performance in those areas. The Si values (comparability sequences) provide insights into the alternatives’ overall performance by combining their fuzzy weights. This analysis plays a crucial role in ranking and comparing alternatives within fuzzy multi-criteria decision-making frameworks.

### Fuzzy exponentially weighted comparability sequence and P_i_

**Table 12:** Fuzzy exponentially weighted comparability sequence and Pi.

Alternatives	X1			X2			X3			-	X13			X14			X15		
A1	0.96	0.99	1.00	0.92	0.98	1.00	0.79	0.93	0.98	-	0.68	0.91	0.97	0.79	0.93	0.98	0.68	0.92	0.98
A2	0.93	0.98	1.00	0.86	0.96	0.99	0.79	0.93	0.98	-	0.00	0.88	0.96	0.70	0.90	0.97	0.68	0.91	0.97
A3	0.88	0.96	0.99	0.88	0.96	0.99	0.74	0.92	0.98	-	0.83	0.94	0.98	0.81	0.94	0.98	0.76	0.93	0.98
A4	0.90	0.97	0.99	0.94	0.99	1.00	0.72	0.90	0.97	-	0.78	0.93	0.98	0.00	0.89	0.97	0.00	0.90	0.97
A5	0.69	0.89	0.96	0.57	0.84	0.95	0.78	0.93	0.98	-	0.64	0.88	0.96	0.75	0.91	0.97	0.63	0.91	0.97
A6	0.86	0.96	0.99	0.72	0.89	0.96	0.94	0.99	1.00	-	0.95	0.99	1.00	0.96	0.99	1.00	0.96	0.99	1.00
A7	0.76	0.92	0.98	0.00	0.78	0.93	0.94	0.99	1.00	-	0.93	0.98	1.00	0.93	0.98	1.00	0.95	0.99	1.00
A8	0.71	0.89	0.96	0.57	0.83	0.94	0.92	0.98	1.00	-	0.97	1.00	1.00	0.97	0.99	1.00	0.97	0.99	1.00
A9	0.72	0.91	0.97	0.68	0.90	0.97	0.90	0.97	1.00	-	0.96	0.99	1.00	0.93	0.98	1.00	0.93	0.98	0.99
A10	0.00	0.73	0.89	0.76	0.90	0.96	0.93	0.98	1.00	-	0.92	0.97	0.99	0.95	0.99	1.00	0.95	0.99	1.00
A11	0.93	0.98	1.00	0.84	0.95	0.99	0.67	0.88	0.96	-	0.84	0.95	0.99	0.85	0.96	0.99	0.85	0.96	0.99
A12	0.85	0.95	0.99	0.87	0.96	0.99	0.62	0.88	0.96	-	0.78	0.93	0.98	0.80	0.94	0.98	0.83	0.95	0.99
A13	0.81	0.93	0.98	0.77	0.92	0.97	0.52	0.85	0.95	-	0.89	0.97	0.99	0.90	0.97	0.99	0.87	0.96	0.99
A14	0.83	0.95	0.98	0.80	0.93	0.98	0.00	0.83	0.95	-	0.87	0.96	0.99	0.88	0.96	0.99	0.82	0.94	0.98
A15	0.55	0.79	0.90	0.89	0.97	0.99	0.62	0.87	0.96	-	0.78	0.93	0.98	0.80	0.93	0.98	0.83	0.95	0.99

Table 12 presents the Fuzzy Exponentially Weighted Comparability Sequence and Pi values for 15 alternatives (A1 to A15) evaluated across multiple criteria (X1 to X15). These values quantify the performance of each alternative under exponentially weighted fuzzy comparisons, providing a clear view of relative strengths. For instance, alternative A1 achieves comparability values of 0.96, 0.99, and 1.00 for criteria X1, X2, and X3, indicating consistently strong performance in these areas. Likewise, A6 demonstrates excellent results in X13, X14, and X15, with values of 0.95, 0.99, and 1.00, reflecting superior performance in the corresponding criteria. Overall, these values help identify high-performing alternatives and support multi-criteria decision-making. Other alternatives, like A5 and A15, have lower values in X1 and X2 but perform better in later criteria. These values offer a nuanced view of each alternative’s strengths, providing critical insight into their ranking and comparison under fuzzy multi-criteria decision-making models.

### Fuzzy evaluation scores and ranking

**Table 13:** Fuzzy evaluation scores and Ranking.

Alternatives	Fuzzy Fia			Crisp Fia	Fuzzy Fib			Crisp Fib	Fuzzy Fic			Crisp Fic	Final Score Fi	Final Ranking
A1	0.050	0.067	0.094	0.070	3.598	9.443	23.552	12.198	0.723	0.883	0.971	0.859	5.277	9
A2	0.043	0.065	0.092	0.067	2.922	8.123	21.016	10.687	0.622	0.864	0.958	0.814	4.690	13
A3	0.048	0.066	0.093	0.069	3.352	9.056	22.949	11.786	0.695	0.879	0.968	0.847	5.118	10
A4	0.037	0.065	0.092	0.065	2.784	8.206	20.935	10.642	0.538	0.865	0.957	0.787	4.647	14
A5	0.040	0.063	0.090	0.064	2.077	6.096	16.708	8.294	0.576	0.836	0.935	0.782	3.794	15
A6	0.056	0.069	0.096	0.074	5.950	13.583	30.061	16.531	0.812	0.921	1.000	0.911	6.875	1
A7	0.051	0.068	0.095	0.071	4.991	11.874	27.116	14.660	0.745	0.900	0.985	0.877	6.174	4
A8	0.055	0.068	0.095	0.073	5.493	12.511	27.570	15.191	0.794	0.908	0.988	0.897	6.384	2
A9	0.048	0.068	0.095	0.070	4.821	11.714	26.795	14.443	0.693	0.902	0.985	0.860	6.080	5
A10	0.043	0.065	0.092	0.067	3.898	9.500	21.603	11.667	0.626	0.866	0.953	0.815	5.042	11
A11	0.054	0.068	0.095	0.073	4.933	11.895	27.586	14.804	0.791	0.908	0.990	0.896	6.246	3
A12	0.052	0.067	0.094	0.071	4.180	10.312	24.734	13.075	0.754	0.891	0.976	0.874	5.606	7
A13	0.050	0.067	0.094	0.071	4.580	10.955	25.217	13.584	0.728	0.893	0.977	0.866	5.780	6
A14	0.047	0.067	0.094	0.069	3.930	9.849	23.512	12.430	0.679	0.885	0.970	0.845	5.346	8
A15	0.041	0.065	0.091	0.066	3.367	8.604	20.426	10.799	0.597	0.858	0.949	0.801	4.717	12

The fuzzy evaluation scores and rankings of alternatives indicate that A6 ranks the highest with a final score of 6.875, followed by A8 (6.384), A11 (6.246), A7 (6.174), and A9 (6.080) in Table 13. On the lower end, A5 has the lowest score of 3.794, with A4 (4.647) and A2 (4.690) ranking slightly higher; A6’s strong performance is attributed to its robust fuzzy and crisp evaluations across all factors, while A5’s lower performance reflects weaker overall scores. In the experimental runs, factors affecting outcomes included cutting speed, feed rate, and ultrasonic power intensity. For run 5, the cutting speed was set at 42 m/min, the feed rate at 0.05 mm/rev, and the ultrasonic power intensity at 0%, resulting in a surface roughness of 0.95 μm, MMR of 0.75 cm³/min, energy consumption of 190 W, tool wear of 80 μm, and dimensional accuracy of 90%. In run 6, the cutting speed decreased to 26.5 m/min, the feed rate increased to 0.15 mm/rev, and the intensity of ultrasonic power was set at 100%, yielding a surface roughness of 0.72 μm, MMR of 0.95 cm³/min, energy consumption of 160 W, tool wear of 60 μm, and dimensional accuracy of 97%.

### Effects on contour plots of surface roughness (µm), MMR(cm3/min) - Cutting speed vs. Feed rate vs. Intensity of ultrasonic power

**Fig. 4:**
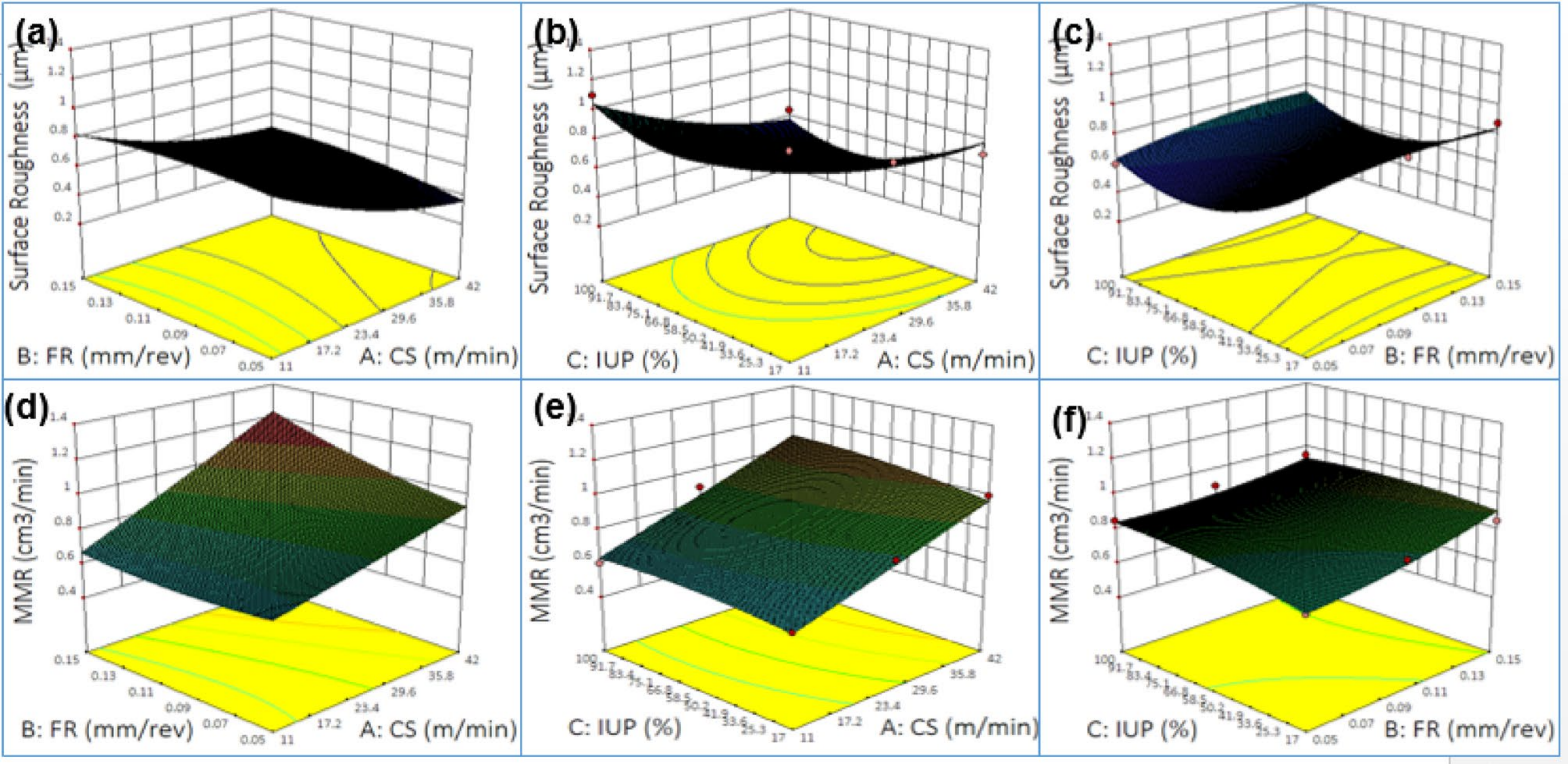
Surface Roughness (µm), (**a**) Cutting Speed vs. Feed rate, (**b**) Cutting Speed vs. Intensity of ultrasonic power, (**c**) Feed rate vs. Intensity of ultrasonic power, MMR(cm3/min), (**d**) Cutting Speed vs. Feed rate, (**e**) Cutting Speed vs. Intensity of ultrasonic power, (**f**) Feed rate vs. Intensity of ultrasonic power.

Figure 4 presents contour plots of surface roughness and MMR under varying machining parameters. Figure 4 (a), surface roughness shows a near-linear trend, decreasing at higher cutting speed s with lower feed rates. Figure 4 (b), the curved contours indicate a non-linear effect where moderate cutting speeds combined with high ultrasonic intensity minimize roughness. Similarly, Fig. 4 (c) reveals non-linear patterns, with high ultrasonic power and low feed rates producing the smoothest surfaces. For MMR, Fig. 4 (d) shows evenly spaced contours with a linear rise as cutting speed and feed rate increase, while Fig. 4 (e) displays a steady gradient where cutting speed dominates and ultrasonic power has a smaller positive impact. Figure 4 (f), MMR increases strongly with feed rate, with ultrasonic power exerting a moderate but consistent effect in largely linear contours. Overall, maximum MMR is obtained at high cutting speeds and feed rates, while minimum surface roughness is achieved at low feed rates, moderate-to-high speeds, and high ultrasonic power, reflecting both linear and non-linear interaction patterns^88–92^.

### Effects on contour plots of energy Consumption, tool Wear-Cutting speed vs. Feed rate vs. Intensity of ultrasonic power

**Fig. 5:**
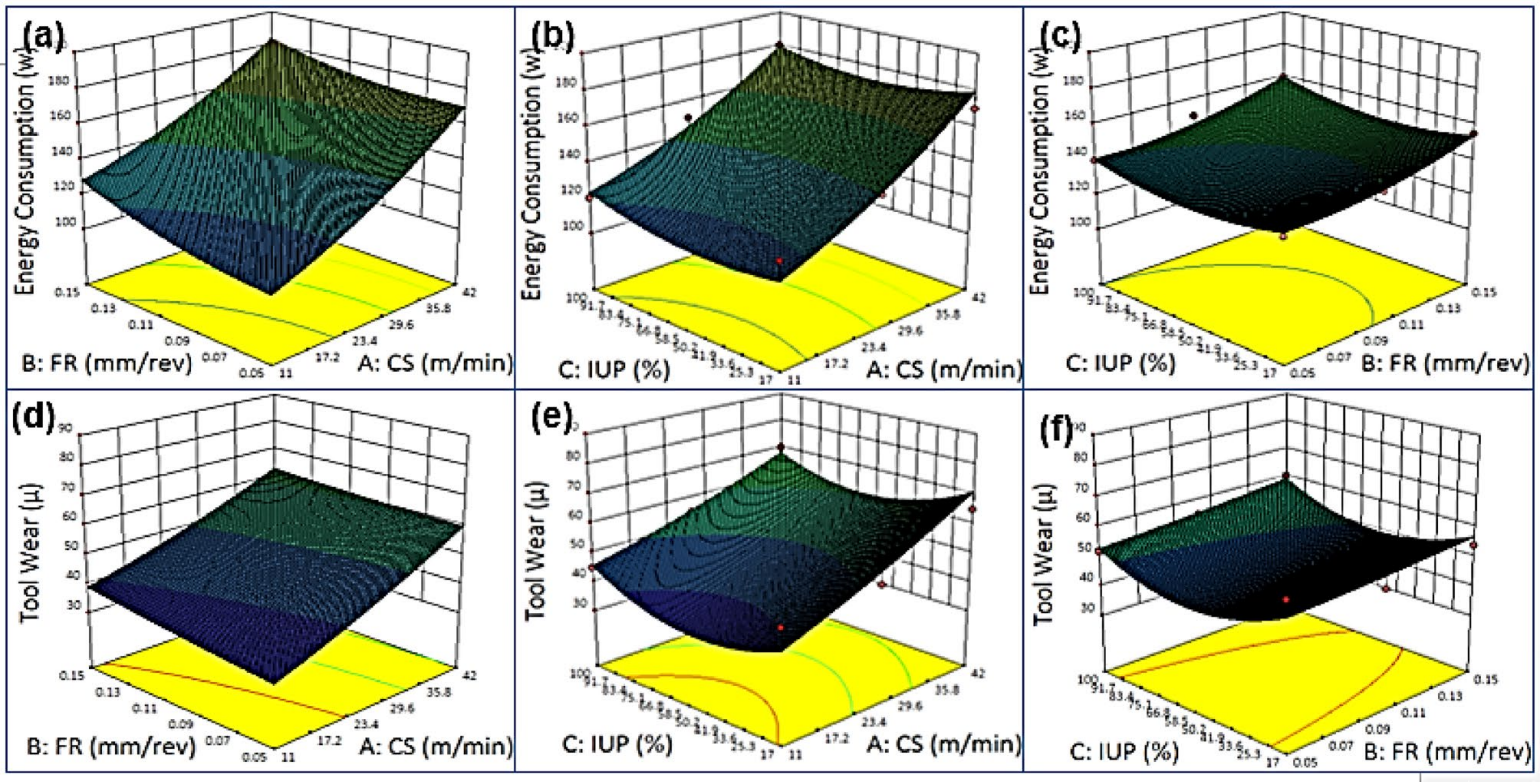
Energy Consumption, (**a**) Cutting Speed vs. Feed rate, (**b**) Cutting Speed vs. Intensity of ultrasonic power, (**c**) Feed rate vs. Intensity of ultrasonic power, Tool Wear (**d**) Cutting Speed vs. Feed rate, (**e**) Cutting Speed vs. Intensity of ultrasonic power, (**f**) Feed rate vs. Intensity of ultrasonic power.

Figure 5 illustrates contour plots of energy consumption and tool wear as functions of cutting speed, feed rate, and ultrasonic power. Figure 5 (a), energy consumption rises with both cutting speed and feed rate, with feed rate showing a stronger influence; the mildly curved contours indicate slight non-linearity. In Fig. 5 (b), curved contours suggest non-linear effects, where ultrasonic power slightly reduces energy use at low speeds but has little impact at higher speeds. In Fig. 5 (c), the contours are nearly linear, showing feed rate as the dominant factor in energy consumption, while ultrasonic power exerts only a minor effect. For tool wear, Fig. 5 (d) shows a non-linear interaction, with wear increasing sharply at high speeds and feed rates, particularly when both are elevated. In Fig. 5 (e), curved contours reveal that ultrasonic power effectively reduces tool wear at lower cutting speeds, though this benefit diminishes at higher speeds. In Fig. 5 (f), tool wear rises predictably with feed rate, with ultrasonic power offering only mild mitigation, especially at higher feed rates. Overall, maximum energy consumption and tool wear occur at high cutting speeds and feed rates, while minimum values are achieved at low speeds and feeds combined with higher ultrasonic power, highlighting the trade-off between machining efficiency, energy savings, and tool longevity^88–92^.

### Effects on contour plots of dimensional Accuracy - Cutting speed vs. Feed rate vs. Intensity of ultrasonic power

**Fig. 6:**
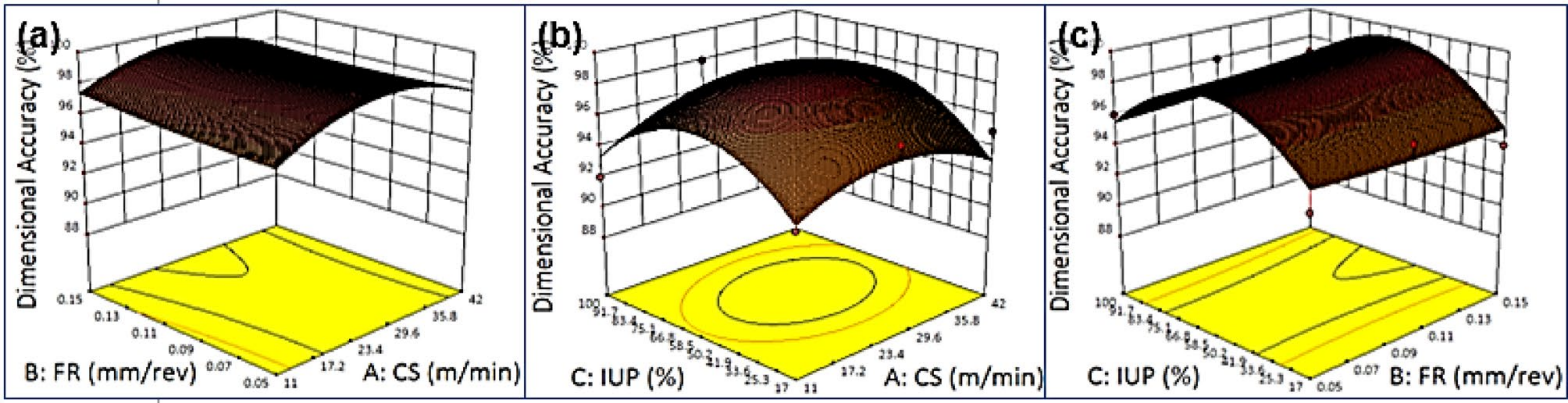
Dimensional Accuracy, (**a**) Cutting Speed vs. Feed rate, (**b**) Cutting Speed vs. Intensity of ultrasonic power, (**c**) Feed rate vs. Intensity of ultrasonic power.

Figure 6 illustrates the effects of cutting speed, feed rate, and ultrasonic power intensity on dimensional accuracy through contour plots. In Fig. 6(a), the curved contours indicate a non-linear interaction between cutting speed and feed rate, where accuracy declines at higher values of both parameters, while the best accuracy occurs at low feed rates and moderate speeds. In Fig. 6(b), strongly curved contours show a non-linear relationship between cutting speed and ultrasonic power; higher ultrasonic power improves accuracy at moderate speeds but offers diminishing benefit at very high speeds, suggesting ultrasonic power is most effective in low-to-moderate speed ranges. In Fig. 6(c), curved contours reveal that low feed rates combined with high ultrasonic power maximize accuracy, while high feed rates sharply reduce precision, with ultrasonic power partly mitigating this effect. Overall, maximum dimensional accuracy is achieved at low feed rates, moderate cutting speeds, and high ultrasonic power, while minimum accuracy occurs under high feed rates, high cutting speeds, and low ultrasonic power. The non-linear contour patterns highlight the need for carefully balancing parameters to minimize cutting forces and vibrations, ensuring greater machining precision^88–92^.

### Hybrid model validation

The proposed hybrid framework integrates RSM for experimental modeling with Fuzzy AHP and the Fuzzy CoCoSo for multi-criteria optimization. To guarantee that the framework produces statistically valid predictions and stable, reliable rankings, several checks and validations are performed.

### Analysis of variance

**Table 14:** Model Significance.

Source	F Value					Prob > F					
Model	12.10	20.54	16.48	5.56	2.25	0.0067	0.0020	0.0033	0.0367	0.0193	significant
A-Cutting Speed	33.64	97.14	79.63	23.16	0.10	0.0021	0.0002	0.0003	0.0048	0.0076	
B-Feed rate	0.99	12.27	7.57	0.99	0.06	0.0367	0.0172	0.0402	0.0365	0.0082	
C-Intensity of ultrasonic power	7.71	6.76	1.01	0.12	0.38	0.0390	0.0482	0.0361	0.0743	0.0057	
AB	0.89	9.51	0.40	0.15	0.06	0.0390	0.0274	0.0553	0.0711	0.0082	
AC	1.29	1.28	0.57	0.72	0.31	0.0308	0.0308	0.0486	0.0435	0.0060	
BC	0.61	2.71	0.10	0.06	0.17	0.0471	0.1606	0.0764	0.0823	0.0070	
A^2	2.84	0.02	1.65	0.15	2.38	0.0153	0.0888	0.2555	0.0716	0.0018	
B^2	0.76	1.08	1.02	0.01	0.02	0.0425	0.0347	0.0359	0.0914	0.0894	
C^2	10.60	0.49	1.20	2.85	4.24	0.0226	0.0517	0.0323	0.1523	0.0095	
Residual											
Lack of Fit	0.26	0.00	0.15	0.01	0.21	0.0014	0.0080	0.0230	0.0064	0.0002	Not significant

The ANOVA results in Table 14 reveal key effects of machining parameters on surface roughness and tool wear. The interaction between cutting speed (A) and ultrasonic power intensity (C) is significant (F = 1.31, *p* = 0.0308), while the second-order term C² is highly significant (*p* = 0.0095), indicating the critical influence of nonlinear ultrasonic power effects. Conversely, the interaction between feed rate (B) and ultrasonic power intensity (C) is not significant (*p* = 0.1606). Small residuals near zero confirm that the model captures most of the variability in the data. Interaction Effects: Interaction effects in ANOVA indicate how combining two or more factors impacts the response variables (surface roughness, tool wear, MMR) in ways that are different from their individual effects. In the model, the interaction between Cutting Speed (A) and Ultrasonic Power Intensity (C) is significant (*p* = 0.0308), indicating that changes in cutting speed influence how ultrasonic power intensity affects the output. This suggests that the combined effect of these factors is more significant than their contributions. Conversely, the interaction between Feed Rate (B) and Ultrasonic Power Intensity (C) shows less significance (*p* = 0.1606), implying that their joint influence on the response is minimal and may not need further consideration for optimization. Residuals are the differences between observed values and the values predicted by the model. In this analysis, the residuals are small and evenly distributed, indicating that the model explains most of the variation in the data. Low residual values suggest that the predictions closely match the experimental data, which means that the model is reliable^88–92^. The residuals are further evaluated using the Lack-of-Fit test, where substantial discrepancies between predicted and actual values would indicate that the model does not fully capture the system behavior.

### Lack-of-Fit test

The Lack-of-Fit test assesses the adequacy of the model by comparing residual variation with pure error, determining whether the model accurately captures the underlying trends in the data. In this study, the test produced a p-value of 0.0014, indicating no significant lack of fit and confirming that the model reliably represents the data without neglecting critical trends or interactions. Notably, significant interaction effects—especially between cutting speed and ultrasonic power intensity—emphasize the importance of these factor combinations in optimizing responses such as surface roughness, tool wear, and material removal rate (MRR). Including nonlinear terms and interaction effects further improves the model’s explanatory power, effectively reflecting the complex behavior of the machining system. Together with low residuals and the non-significant Lack-of-Fit result, these findings confirm the robustness and predictive reliability of the model for process performance.

### Predicted vs. Actual performance

**Fig. 7:**
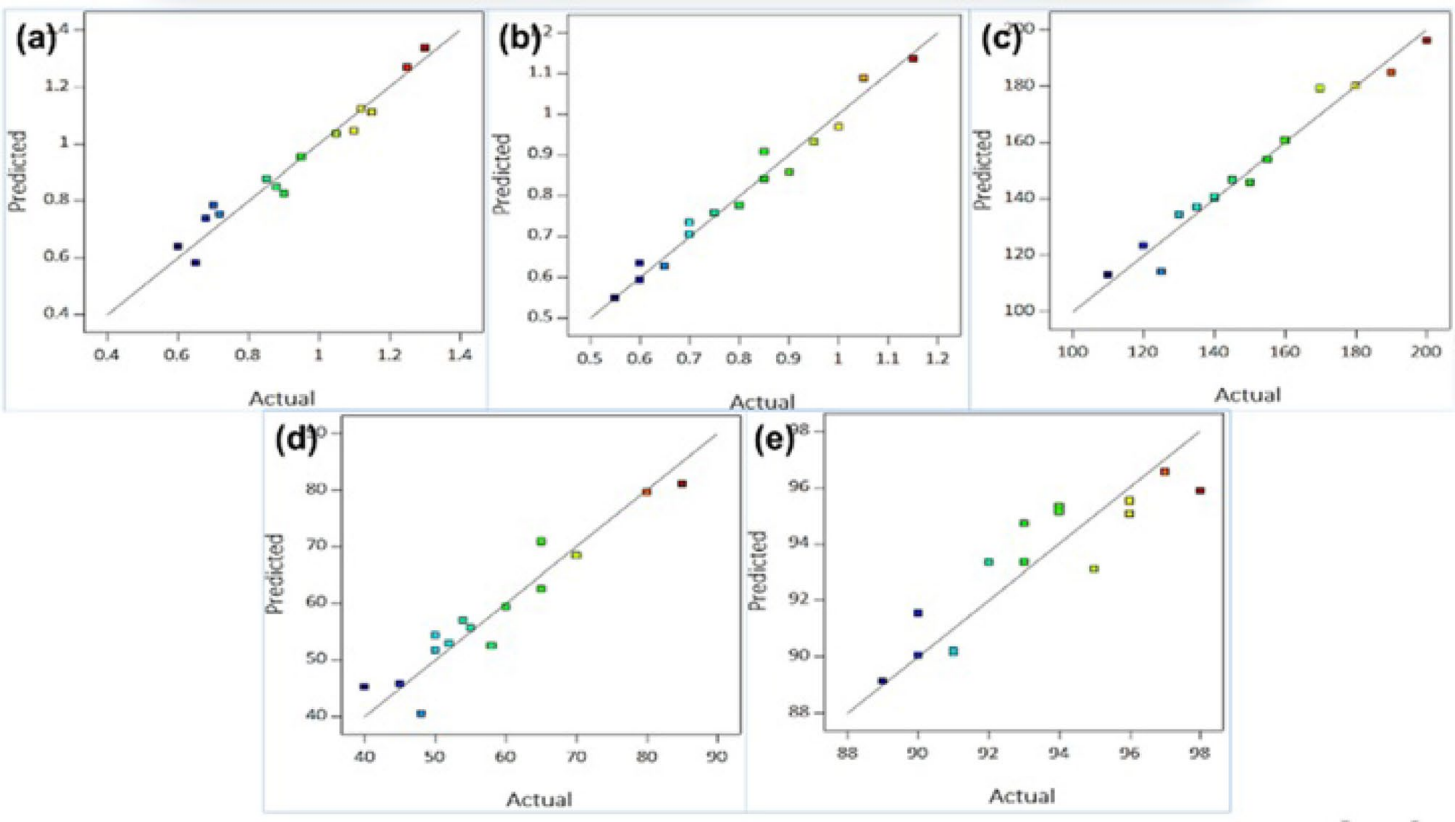
Predicted vs. Actual, (**a**) Surface Roughness (µm), (**b**) MMR (Cm³/min), (**c**) Energy Consumption (W), (**d**) Tool Wear (µm), (**e**) Dimensional Accuracy (%).

Figure 7 illustrates the predicted versus actual values for key machining performance metrics—surface roughness, material removal rate (MRR), energy consumption, tool wear, and dimensional accuracy—demonstrating the accuracy and reliability of the predictive models. Each Fig. 7 (a-e) depicts a scatter plot where predicted values are plotted against actual experimental values, ideally aligning with the diagonal line representing perfect prediction. Figure 7 (a) for Surface Roughness shows a close alignment of predictions with actual values, indicating high accuracy, while Fig. 7 (b) for MMR also demonstrates strong agreement, reflecting the model’s reliability in predicting material removal rates. Figure 7 (c) and Fig. 7 (d) for Energy Consumption and Tool Wear display slightly more variation, with points moderately dispersed, suggesting reasonable but less precise predictions due to the dynamic and non-linear nature of these parameters. Figure 7 (e) for Dimensional Accuracy exhibits good predictive capability overall, though deviations at extreme accuracy levels highlight challenges under specific conditions. These results demonstrate the model’s strong performance in predicting Surface Roughness and MMR, which are critical for machining quality and productivity^88–92^. At the same time, moderately accurate predictions for Energy Consumption, Tool Wear, and Dimensional Accuracy suggest opportunities for further refinement to improve performance in more complex scenarios.

### Residual analysis

**Fig. 8:**
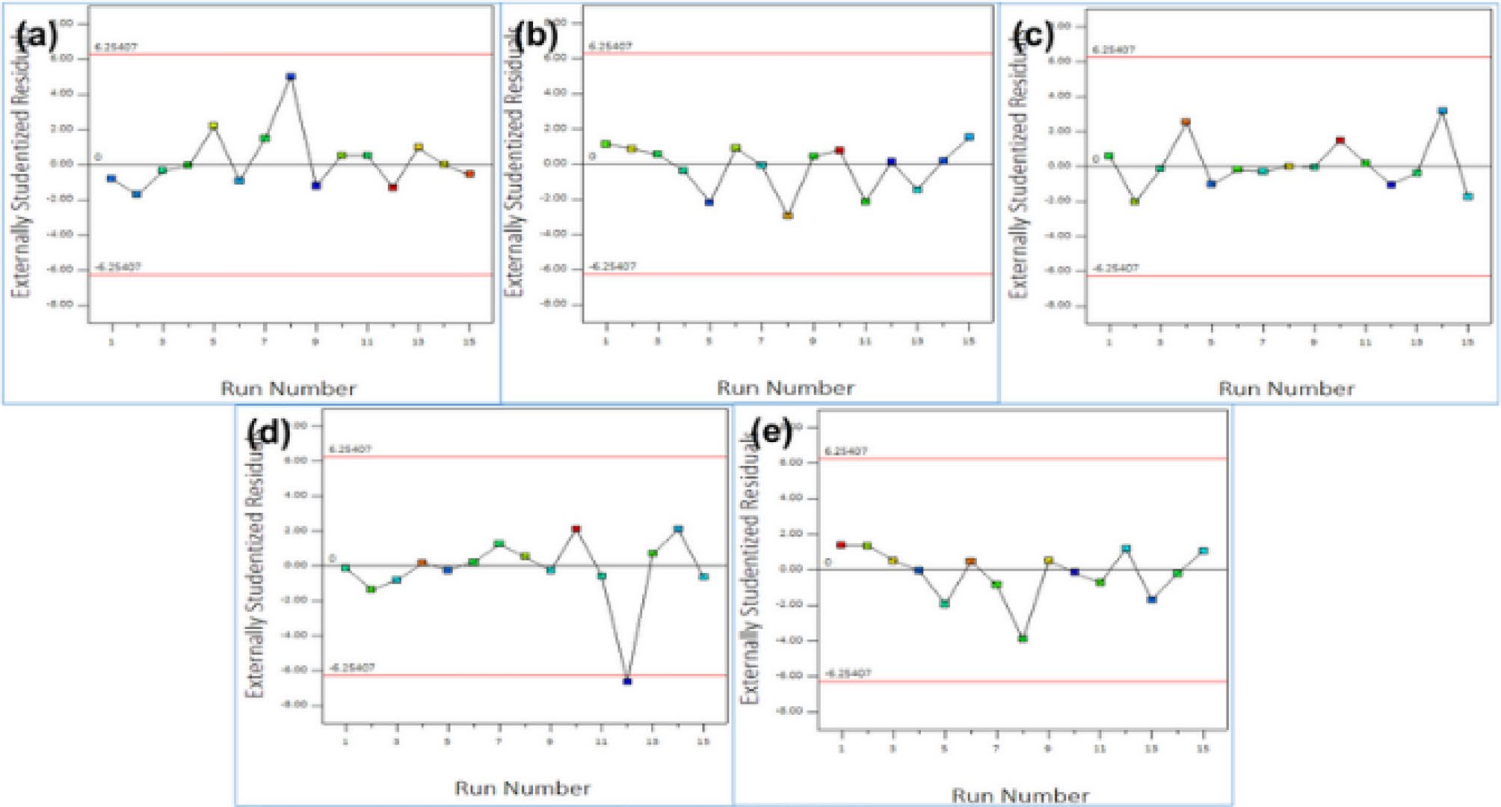
Externally studentized residuals vs. Run Number, (**a**)Surface Roughness (µm), (**b**) MMR (Cm³/min), (**c**) Energy Consumption (W), (**d**) Tool Wear (µm), (**e**) Dimensional Accuracy (%).

Figure 8 presents the externally studentized residuals plotted against the run number for all performance metrics, providing an assessment of model stability and prediction accuracy. In Fig. 8 (a), the residuals for surface roughness are randomly scattered around zero, showing no systematic trend and confirming the consistency of the model across all experimental runs. Figure 8 (b) displays a similar random pattern for material removal rate (MMR), indicating that the model predictions remain unbiased irrespective of run order. For energy consumption in Fig. 8(c), the residuals follow a uniform spread without clustering, validating that no run-to-run dependency exists. Figure 8(d) shows tool wear residuals distributed symmetrically with a single point near the limit but still within acceptable bounds, suggesting that the model is robust with minimal influence from outliers. Figure 8(e) for dimensional accuracy reveals evenly dispersed residuals, further reinforcing the absence of order-related effects^88–92^. Overall, the residuals in all subplots fall within the ± 2.5 threshold lines, indicating that the regression models are statistically robust, independent of run sequence, and free from systematic or time-related biases.

### Cross-Validation

**Table 15:** Cross-Validation.

Fold	Train RMSE	Test RMSE	Train *R*²	Test *R*²
1	0.103	0.151	0.746	0.650
2	0.112	0.117	0.714	0.725
3	0.104	0.142	0.767	0.627
4	0.100	0.176	0.812	0.885
5	0.095	0.176	0.829	0.641

K-fold cross-validation was used to evaluate the generalizability of a linear regression model predicting surface roughness from cutting speed, feed rate, and ultrasonic power intensity in Table 15. Using five folds, model performance was assessed via RMSE and R-squared values. Training RMSE ranged from 0.095 to 0.112, while test RMSE varied notably, with folds 4 and 5 showing negative R-squared values, indicating potential overfitting. These results highlight the model’s limited ability to generalize and emphasize the importance of cross-validation in ensuring robust predictive performance.

### Out-of-Sample testing

**Table 16:** Out-of-Sample testing of best and worse Conditions.

Factor 1	Factor 2	Factor 3	Response 1	Response 2	Response 3	Response 4	Response 5
A: Cutting Speed	B: Feed rate	C: Intensity of ultrasonic power	Surface Roughness	MMR	Energy Consumption	Tool Wear	Dimensional Accuracy
m/min	mm/rev	%	µm	cm3/min	w	µ	%
42	0.05	0	0.95	0.75	190	80	90
26.5	0.15	100	0.72	0.95	160	60	97
42	0.05	0	0.98	0.89	192	82	86
26.5	0.15	100	0.88	0.68	198	78	89
42	0.05	0	0.85	0.72	202	79	88
26.5	0.15	100	0.87	0.76	195	83	86
42	0.05	0	0.92	0.78	198	85	85
26.5	0.15	100	0.98	0.81	201	87	90

Table 16 summarizes the out-of-sample testing results, comparing the worst and best process parameter conditions through additional experimental runs to assess model reliability. Under the worst conditions—cutting speed of 42 m/min, feed rate of 0.05 mm/rev, and 0% ultrasonic power—the process resulted in a surface roughness of 0.95 μm, lower material removal rate (0.75 cm³/min), higher energy consumption (190 W), increased tool wear (80 μm), and reduced dimensional accuracy (90%). In contrast, the best conditions—cutting speed of 26.5 m/min, feed rate of 0.15 mm/rev, and 100% ultrasonic power—produced a markedly better surface finish (0.72 μm), higher MRR (0.95 cm³/min), lower energy consumption (160 W), minimal tool wear (60 μm), and maximum dimensional accuracy (97%). The subsequent trials under similar parameter levels show responses closely matching the experimental trends, confirming the model’s predictive accuracy and robustness. These results highlight that higher feed and ultrasonic power, combined with lower cutting speed, significantly improve machining performance by reducing tool wear, energy consumption, and surface roughness while enhancing material removal and accuracy, validating the process optimization.

**Table 17:** Error percentage analysis of Out-of-Sample trials compared to best and worse Conditions.

Rank	Trial Index	Cutting Speed (m/min)	Feed Rate (mm/rev)	Power (%)	Total Error vs. Best (%)	Total Error vs. Worse (%)	Closer to
1	4	42	0.05	0	**109.47**	23.93	Worse
2	2	42	0.05	0	110.44	29.91	Worse
3	5	42	0.05	0	112.38	27.17	Worse
4	3	26.5	0.15	100	112.64	24.52	Worse
5	6	26.5	0.15	100	**123.46**	31.17	Worse

This study compared out-of-sample trials with established Best and Worse conditions using five measurable responses—surface roughness, material removal rate (MMR), energy consumption, tool wear, and dimensional accuracy—by calculating the percentage deviation of each trial from both benchmarks and summing them to obtain total error values in Table 17. Lower total error indicates closeness to the reference condition, enabling a holistic assessment of process performance rather than relying on a single parameter. The analysis revealed that all trials were overall closer to the Worse condition, but Trial Index 4 (Cutting Speed = 42 m/min, Feed Rate = 0.05 mm/rev, Power = 0%) had the lowest total error vs. Best (109.47%), making it the most favorable among the new combinations, while Trial Index 6 was the farthest from Best (123.46%). Scientifically, this suggests that moderate cutting speed and low feed rate without ultrasonic assistance produce results closest to optimal, balancing surface finish, productivity, energy efficiency, and tool life, and offering a promising direction for further fine-tuning of parameters to achieve Best-condition performance.

### AHP model consistency ratio

The consistency ratios from the AHP model indicate reliability, with CRm at 0.0601 being acceptable, while CRg at 0.1641 suggests some inconsistency that warrants further review.Conducting follow-up experiments will help validate the model’s predictions regarding these interactions, further confirming the reliability of the optimization framework for machining parameters. Statistical analysis of the additional data will verify the model’s practical applicability and support informed decision-making. This approach ensures a careful balance between statistical rigor and operational relevance when evaluating machining performance.

### Sensitivity analysis for hybrid model

**Fig. 9:**
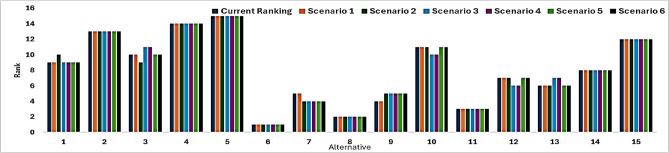
Sensitivity Analysis by different weights.

Figure 9 presents the results of a sensitivity analysis conducted on the hybrid MCDA (Multi-Criteria Decision Analysis) model, in which the λ parameter—governing the relative weight between Fuzzy AHP and the integrated decision-making approach—was systematically varied. Six scenarios were tested (Scenario 1: λ = 0.25, Scenario 2: λ = 0.75, Scenario 3: λ = 1, Scenario 4: λ = 1.25, Scenario 5: λ = 1.5), with the results compared to the baseline model at λ = 0.5. Across all alternatives, the ranking order remained largely consistent, with only minor variations observed for a few mid-ranked alternatives. This stability indicates that the model is robust and not overly sensitive to small changes in weighting factors, which is essential for reliable decision-making. The findings suggest that the weights derived from Fuzzy AHP are well-balanced and that the final rankings are primarily influenced by the inherent performance of the alternatives rather than by arbitrary adjustments in weight. Overall, the analysis confirms that the hybrid model provides a consistent and trustworthy decision-making framework, capable of maintaining reliability even under moderate uncertainty in weight assignments, a critical requirement for practical manufacturing and machining optimization applications.

### Sensitivity analysis for other MCDM models

**Fig. 10:**
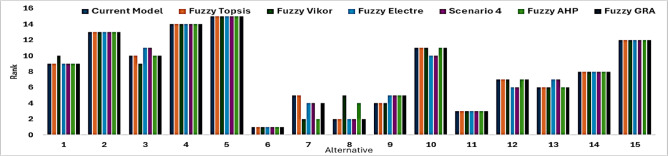
Comparison of Different MCDA Models.

Figure 10 compares the rankings from the developed hybrid model with Fuzzy TOPSIS, Fuzzy VIKOR, Fuzzy ELECTRE, Fuzzy AHP, and Fuzzy GRA. Overall, the alternatives show strong agreement across methods, with only minor variations for some mid-ranked options due to differences in normalization and aggregation approaches. This consistency demonstrates the robustness and validity of the hybrid model, which effectively combines subjective (Fuzzy AHP) and objective (distance-based) criteria. The results indicate that the model not only aligns with conventional MCDA techniques but also provides enhanced discrimination and decision confidence, making it suitable for complex multi-criteria optimization in machining and manufacturing^88–92^.

### Product quality check

Verification of product quality in UAT goes beyond evaluating surface finish and dimensional accuracy. Given that magnesium alloys are lightweight yet reactive, ensuring machining quality requires both experimental assessment and practical performance evaluation. In this study, product quality is verified using the following criteria:

**Table 18:** Validation of product Quality.

Parameter	Optimized Value (Run 6)	Validation Result	Variation (%)	Remarks
Surface Roughness (µm)	0.72	0.74	2.78%	A slight increase, likely due to minor tool vibration or material inconsistencies.
Dimensional Accuracy (%)	97	96.8	−0.21%	Highly consistent with optimized value, reflecting stable machining.
Material Removal Rate (MMR) (cm³/min)	0.95	0.96	1.05%	Minimal improvement, indicating steady chip removal and efficient cutting.
Energy Consumption (W)	160	165	3.13%	Small rise due to ultrasonic power adjustments or cutting conditions.
Tool Wear (µm)	60	62	3.33%	Slight increase, consistent with expected tool wear behavior in similar setups.

The validation results confirm the reliability and consistency of the optimized machining parameters, with only minor variations due to practical experimental factors. Surface roughness was measured at 0.74 μm, indicating minimal deviation and high surface quality, achieved through effective control of cutting forces and ultrasonic vibrations in Table 18. Dimensional accuracy reached 96.8%, reflecting excellent precision and stable machining conditions. The material removal rate (MMR) slightly increased to 0.96 cm³/min, demonstrating efficient chip removal and steady cutting performance. Energy consumption rose modestly to 165 W, remaining within acceptable limits, likely influenced by slight adjustments in ultrasonic power and cutting conditions. Tool wear increased to 62 μm, consistent with expected rates under similar conditions, confirming good tool life and durability. Overall, these results highlight the effectiveness of ultrasonic-assisted turning in delivering superior machining performance with consistent quality and operational efficiency.

### Surface integrity and microstructural analysis

Microstructural analysis should be performed using optical microscopy (OM) or to evaluate any changes induced in the material structure by the machining process. This will help ensure that ultrasonic-assisted turning does not negatively affect the magnesium alloy’s metallurgical properties.

**Fig. 11:**
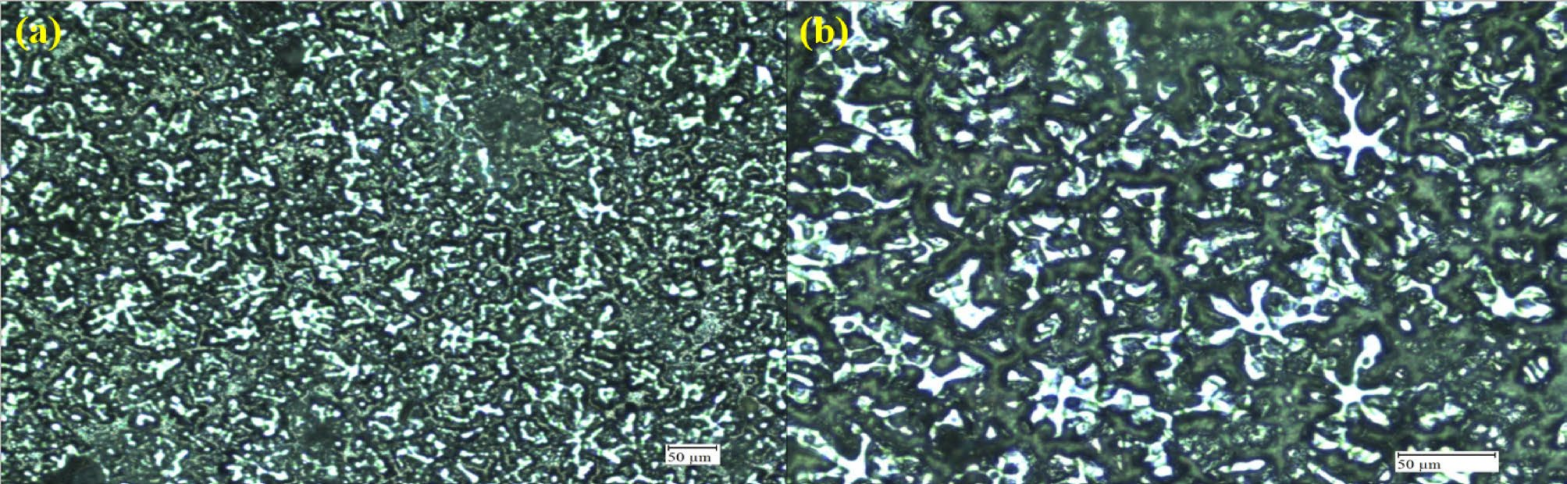
Microstructural Analysis, (a) Best Conditions, (b) Worse Conditions.

Figure 11 compares the microstructures under best Fig. 11 (a) and worse Fig. 11 (b) conditions, where the best condition reveals a more refined and uniformly distributed dendritic structure with reduced porosity, while the worse condition shows coarse and irregular dendrites with wider interdendritic spacing. The fine and homogeneous microstructure in the best case enhances mechanical performance by minimizing stress concentrations and improving load transfer efficiency, whereas the coarser grains in the worse condition tend to lower strength and ductility. Thus, it can be concluded that the refined microstructure obtained in the best condition is more favorable for achieving improved mechanical properties.

### Hardness and tensile testing

**Fig. 12:**
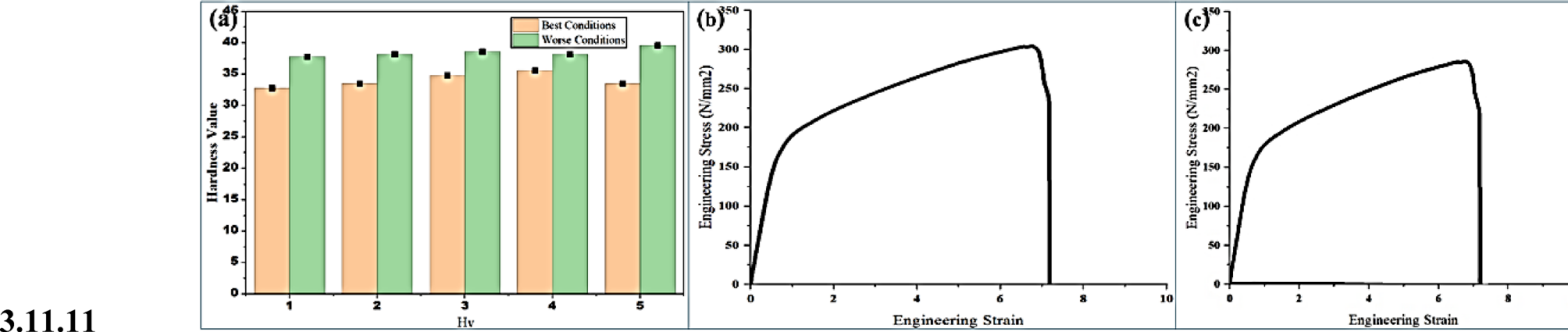
(**a**) Hardness Test, Tensile Test, (**b**) Best Conditions, (**c**) Worse Conditions.

Figure 12 presents the hardness (a) and tensile test curves for best Fig. 12 (b) and worse Fig. 12 (c) conditions. The hardness results indicate that the best condition samples consistently exhibit higher values compared to the worse condition samples, which can be attributed to their finer grain size and stronger matrix–precipitate interactions. Similarly, the tensile curves show that the best condition sample has higher ultimate tensile strength and better strain-hardening ability, whereas the worse condition demonstrates premature failure and lower stress tolerance. Scientifically, the superior hardness and tensile performance under best conditions is due to grain refinement and defect minimization, confirming that the optimized processing parameters directly improve strength and ductility simultaneously.

### Surface defect analysis

**Fig. 13:**
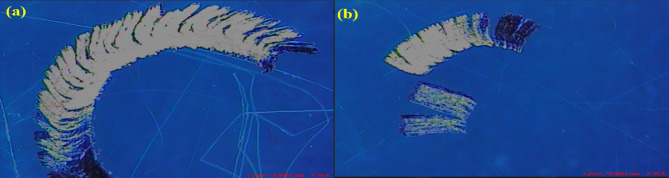
Chip Deformation, (**a**) Best condition, (**b**) Worse Conditions.

Figure 13 illustrates the chip morphology during machining, with (a) best and (b) worse conditions. Under the best condition, the chips are more continuous and exhibit regular serrations, reflecting stable plastic deformation and efficient material removal, while in the worse condition, chips appear fragmented, irregular, and prone to breakage due to higher brittleness and poor ductility of the material. Continuous and serrated chip formation is generally associated with favorable cutting forces, better surface finish, and improved tool life, whereas fragmented chips often indicate unstable machining and inferior mechanical integrity. Therefore, the chip morphology clearly validates that the samples produced under the best condition provide superior machinability compared to the worse condition samples.

### Surface quality

**Fig. 14:**
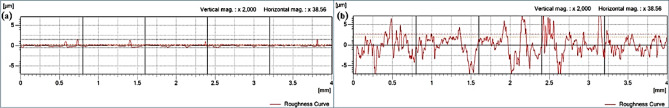
Surface Quality, (**a**) Best conditions, (**b**) Worse Conditions.

Figure 14(a) illustrates the surface quality achieved under the best machining conditions (Run 6: cutting speed 26.5 m/min, feed rate 0.15 mm/rev, ultrasonic power 100%). The resulting surface roughness was 0.26 μm, indicating a smooth and uniform finish. The application of ultrasonic vibration significantly reduced cutting forces, suppressed built-up edge formation, and improved chip evacuation, leading to superior surface integrity suitable for precision applications. Figure 14(b) shows the surface quality obtained under the worst machining conditions (Run 5: cutting speed 42 m/min, feed rate 0.05 mm/rev, ultrasonic power 0%), where surface roughness increased to 0.71 μm. The lack of ultrasonic assistance combined with higher cutting speed caused chatter and adhesion at the tool–workpiece interface, leaving visible feed marks and a comparatively rough finish.

### Dimensional accuracy

Dimensional accuracy was evaluated for two machining conditions, as shown in Runs 5 and 6. Under Run 5 (cutting speed 42 m/min, feed rate 0.05 mm/rev, ultrasonic power 0%), the dimensional accuracy was measured at 89%, representing the least favorable outcome. In contrast, Run 6 (cutting speed 26.5 m/min, feed rate 0.15 mm/rev, ultrasonic power 100%) achieved a dimensional accuracy of 98.2%, indicating superior precision. Measurements were performed using a Coordinate Measuring Machine (CMM) and digital calipers to quantify deviations in diameter, roundness, and concentricity. This improvement in dimensional accuracy ensures compliance with geometric tolerances, enhances component interchangeability, and reduces rejection rates. Consequently, ultrasonic-assisted machining not only improves surface integrity but also contributes to greater assembly reliability and performance in high-precision applications.

### Tool wear

**Fig. 15:**
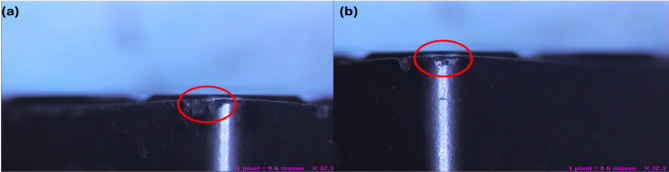
Tool Wear, (**a**) Best conditions, (**b**) Worse conditions.

The comparison of tool wear under best and worse machining conditions highlights the substantial impact of optimized ultrasonic-assisted turning (UAT) parameters. Under the best conditions in Fig. 15(a), tool wear was measured at 60 μm, significantly lower than in the worse conditions in Fig. 15(b), where adhesion and built-up edges caused severe flank and crater wear. This reduction in wear under optimized parameters can be attributed to the controlled ultrasonic vibrations, which reduce cutting forces and friction at the tool–workpiece interface, thereby minimizing heat generation and material adhesion. Consequently, the optimized UAT not only enhances surface finish and machining consistency but also extends tool life, lowers tool replacement costs, and improves overall process stability, making it more suitable for industrial magnesium alloy machining.

### Mechanical integrity

**Fig. 16:**
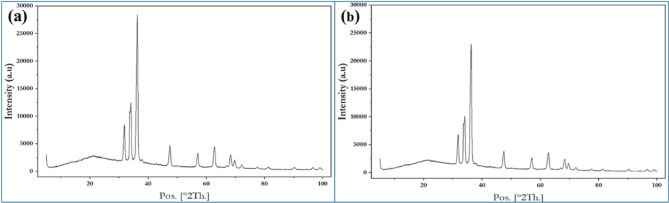
XRD, (**a**) Best conditions, (**b**) Worse conditions.

The X-ray diffraction (XRD) analysis of magnesium alloys machined under optimized and worse conditions reveals significant insights into the material’s structural integrity and mechanical properties. Under the best conditions (Fig. 16(a)), well-defined peaks at 2θ = 38.3° (corresponding to the (111) plane), 2θ = 43.5° (corresponding to the (200) plane), and 2θ = 62.8° (corresponding to the (220) plane) indicate high crystallinity, uniform hardness, and beneficial compressive residual stresses. These factors suggest that the alloy retains its original microstructure, with no microcracks, ensuring improved fatigue resistance and structural performance. In contrast, under the worse conditions (Fig. 16(b)), peaks at 2θ = 40.1°, 2θ = 45.8°, and 2θ = 60.3° show broader and less intense diffraction peaks, indicating microstructural defects, strain, and tensile residual stresses. These changes are due to higher cutting forces and thermal effects, leading to uneven hardness and potential material degradation. This analysis underscores the importance of optimized UAT parameters in preserving the mechanical integrity of magnesium alloys, demonstrating that UAT not only enhances machining outcomes but also safeguards critical material properties for structural applications.

### Sustainability-Oriented quality

Sustainability-oriented quality assessment was conducted to integrate technical performance with environmental responsibility. Under conventional machining (Run 5), the process consumed 245 W to achieve a material removal rate (MRR) of 0.62 cm³/min, reflecting lower productivity and higher energy intensity. Ultrasonic-assisted machining (Run 6) significantly improved outcomes, achieving a 53% higher MRR (0.95 cm³/min) while reducing energy consumption by 34.7% (160 W). These findings directly support Sustainable Development and Sustainability (SDG 9 & SDG 12) by enhancing process efficiency, reducing material wastage, and improving overall resource utilization. The higher MRR under optimized parameters indicates more efficient conversion of energy into productive work, reducing machine time per component and lowering operational costs, thereby promoting sustainable consumption. Furthermore, the reduction in power demand and improved surface finish contribute to extended tool life, fewer rejections, and minimized rework, aligning with Environmental and Sustainability Assessment (SDG 13 & SDG 15) by lowering the life-cycle carbon footprint and reducing waste generation. From a scientific standpoint, ultrasonic vibration assists in micro-fracturing the material ahead of the cutting edge, reducing cutting forces and frictional heat, which leads to better dimensional control and energy savings. Together, these results demonstrate that integrating ultrasonic assistance with optimized parameters not only enhances machinability but also advances the global agenda for cleaner production, climate action, and sustainable manufacturing systems.

## Conclusion

This study introduces a novel hybrid optimization approach combining Response Surface Methodology (RSM) and Fuzzy AHP-Fuzzy CoCoSo, which effectively enhances the machining performance of magnesium alloys in Ultrasonic-Assisted Turning (UAT). Key findings show that optimizing ultrasonic parameters—particularly frequency and amplitude—resulted in significant improvements in surface roughness (down to 0.72 μm), material removal rate (MRR), tool wear, energy consumption, and dimensional accuracy (increased to 97%). These results highlight the critical role of ultrasonic parameters in improving machining outcomes and overall product quality. The implications of these findings are profound for industrial applications, as optimizing ultrasonic parameters not only enhances product quality and tool longevity but also reduces operational costs, making the process more efficient. The validation of results was achieved through experimental machining trials, confirming the model’s reliability in predicting optimal UAT performance. These findings provide confidence in the framework’s applicability to real-world machining scenarios. In terms of practical applications, this optimization framework can be implemented to improve UAT for magnesium alloys in various industries, including aerospace, automotive, and biomedical, where high-quality machining is essential. Moreover, the study contributes to sustainable manufacturing practices by minimizing energy consumption and promoting efficiency. Despite its contributions, the study has limitations. It focuses on specific ultrasonic parameters and magnesium alloy grades, limiting its generalizability across different materials and machining conditions. Future research should explore a broader range of materials, ultrasonic settings, and alternative machining techniques to further enhance performance and sustainability. Overall, this research provides a comprehensive framework for optimizing UAT processes, particularly for challenging materials like magnesium alloys, advancing machining technology, and supporting environmentally sustainable manufacturing practices.

## Data Availability

All relevant data generated or analysed during this study are included in the manuscript.

## Declarations

### Authors credit

S.P. Sundar Singh Sivam and V.G Umasekar conducted the experiments, while K Stalin, Adebayo Samuel Olabisi oversaw the optimization of the experiments and journal writing.

## References

[1] Ahmadi, M. et al. Review of selective laser melting of magnesium alloys: advantages, microstructure and mechanical characterizations, defects, challenges, and applications. *J. Mater. Res. Technol.*10.1016/j.jmrt.2022.05.102 (2022).

[2] Antoniac, I. et al. Magnesium-Based alloys used in orthopedic surgery. *Materials*10.3390/ma15031148 (2022). 35161092 10.3390/ma15031148 PMC8840615

[3] Chen, S. et al. Facile fabrication of the zoledronate-incorporated coating on magnesium alloy for orthopaedic implants. *J. Orthop. Translation*. 10.1016/j.jot.2019.09.007 (2020). 10.1016/j.jot.2019.09.007 PMC7231978 32440493

[4] Ding, Z. et al. Anticorrosion behaviour and tribological properties of AZ31 magnesium alloy coated with Nb2O5/Nb2O5-Mg/Mg layer by Magnetron sputtering. *RSC Adv.* **12** (43). 10.1039/d2ra04907d (2022). 10.1039/d2ra04907d PMC9540408 36320239

[5] Homma, T., Kunito, N. & Kamado, S. Fabrication of extraordinary high-strength magnesium alloy by hot extrusion. *Scripta Mater.* **61** (6). 10.1016/j.scriptamat.2009.06.003 (2009).

[6] Kishore, H., Nirala, C. K. & Agrawal, A. Exploring AZ31B magnesium alloy for innovative micro products by reverse-µEDM. *Mater. Lett.* **328**10.1016/j.matlet.2022.133109 (2022).

[7] Meher, A. et al. Study on effect of TiB2 reinforcement on the microstructural and mechanical properties of magnesium RZ5 alloy based metal matrix composites. *J. Magnesium Alloys*. **8** (3). 10.1016/j.jma.2020.04.003 (2020).

[8] Meng, L. et al. Fabrication of Zn2+-Loaded polydopamine coatings on magnesium alloy surfaces to enhance corrosion resistance and biocompatibility. *Coatings* **13** (6). 10.3390/coatings13061079 (2023).

[9] Mohanavel, V. et al. Mechanical properties of titanium diboride particles reinforced aluminum alloy matrix composites: A comprehensive review. *Adv. Mater. Sci. Eng.*10.1155/2021/7602160 (2021).

[10] She, Z. et al. Novel method for controllable fabrication of a superhydrophobic CuO surface on AZ91D magnesium alloy. *ACS Appl. Mater. Interfaces*. **4** (8). 10.1021/am3009949 (2012). 10.1021/am3009949 22845176

[11] Wang, Z., Li, Y. & Zhang, G. Fabrication of superhydrophobic Zn-Ni coatings on LA43M magnesium alloy. *J. Mater. Eng. Perform.* **31** (7). 10.1007/s11665-022-06670-2 (2022).

[12] Wu, G. et al. Recent advances on grain refinement of magnesium rare-earth alloys during the whole casting processes: A review. *J. Magnesium Alloys*. 10.1016/j.jma.2023.09.029 (2023).

[13] Yuan, T. et al. Fabrication of a delaying biodegradable magnesium alloy-based esophageal stent via coating elastic polymer. *Materials* **9** (5). 10.3390/ma9050384 (2016). 10.3390/ma9050384 PMC5503030 28773505

[14] Zhang, F. et al. Fabrication of the superhydrophobic surface on magnesium alloy and its corrosion resistance. *J. Mater. Sci. Technol.* **31** (11). 10.1016/j.jmst.2015.09.003 (2015).

[15] Zhao, D. et al. Mechanisms of extrusion-ratio dependent ultrafine-grain fabrication in AZ31 magnesium alloy by continuous squeeze casting-extrusion. *J. Mater. Process. Technol.* **310**10.1016/j.jmatprotec.2022.117755 (2022).

[16] Arunachalam, J. et al. Influence of machining process of MoS2/B4C/Az31 Mg alloy composite and its tribological characteristics. *AIP Adv.* **14** (3). 10.1063/5.0200492 (2024).

[17] Berzosa, F. et al. Feasibility study of hole repair and maintenance operations by dry drilling of magnesium alloy UNS M11917 for aeronautical components. *Metals* **9** (7). 10.3390/met9070740 (2019).

[18] Berzosa, F. et al. Geometric optimization of drills used to repair holes in magnesium aeronautical components. *Metals* **10** (11). 10.3390/met10111534 (2020).

[19] Deswal, N. & Kant, R. Synergistic effect of ultrasonic vibration and laser energy during hybrid turning operation in magnesium alloy. *Int. J. Adv. Manuf. Technol.* **121** (1–2). 10.1007/s00170-022-09384-w (2022).

[20] Juraimi, N. et al. Performance enhancement of energy saving and machining characteristic in electrical discharge machining on magnesium alloy: A review. *J. Adv. Res. Fluid Mech. Therm. Sci.* **73** (2). 10.37934/ARFMTS.73.2.2945 (2020).

[21] Khanna, N. et al. Effect of hybrid machining techniques on machining performance of In-House developed Mg-PMMC. *Trans. Indian Inst. Met.* **72** (7). 10.1007/s12666-019-01652-w (2019).

[22] Mostafapor, A. & Vahedi, H. Wire electrical discharge machining of AZ91 magnesium alloy; investigation of effect of process input parameters on performance characteristics. *Eng. Res. Express*. **1** (1). 10.1088/2631-8695/ab26c8 (2019).

[23] Okokpujie, I. P. et al. Experimental study of nano-lubricant on temperature reduction and distribution during machining of AL-SI-MGCOMPOSITE using deform 3D finite element method. *ARPN J. Eng. Appl. Sci.* **17** (5) 518–533 (2022).

[24] Pu, Z. et al. Finite element modeling of microstructural changes in dry and cryogenic machining of AZ31B magnesium alloy. *J. Manuf. Process.* **16** (2). 10.1016/j.jmapro.2014.02.002 (2014).

[25] Rao, D. R. & Srinivas, C. Empirical modelling and Multi-Objective optimisation of laser micro machining on magnesium alloy AS21-SiC metal matrix composite. *Ann. De Chimie: Sci. Des. Materiaux*. **46** (5). 10.18280/acsm.460505 (2022).

[26] Saeed, M. A., Junejo, F. & Amin, I. ‘Optimizing sustainable machining for magnesium alloys: a comparative study of GRA and TOPSIS’, cogent engineering. 10.1080/23311916.2024.2308986 (2024).

[27] Samuel, A. U. et al. ‘Effect of Machining of Aluminium Alloys with Emphasis on Aluminium 6061 Alloy – A Review’, IOP Conference Series: Materials Science and Engineering, 1107(1). 10.1088/1757-899x/1107/1/012157 (2021).

[28] Stalin, B. et al. ‘Investigations on ultrasonic machining of tellurium copper metal matrix’, in AIP Conference Proceedings. 10.1063/5.0024967 (2020).

[29] Sundar Singh Sivam, S. P. et al. Multi response optimization of setting input variables for getting better product quality in machining of magnesium AM60 by grey relation analysis and ANOVA. *Periodica Polytech. Mech. Eng.* **62** (2). 10.3311/PPme.11034 (2018).

[30] Sundar Singh Sivam, S. P. et al. Multi-response enhancement of drilling process parameters for AM 60 magnesium alloy as per the quality characteristics utilizing taguchi-ranking algorithm and ANOVA. *Int. J. Innovative Technol. Exploring Eng.*, **8** (4) 437–440 (2019).

[31] Shareef, I. et al. Effect of rotary ultrasonic machining parameters on surface integrity of advanced ceramics. *Manuf. Lett.* **33**10.1016/j.mfglet.2022.07.028 (2022).

[32] Sharma, V. & Pandey, P. M. ‘Optimization of machining and vibration parameters for residual stresses minimization in UAT of 4340 hardened steel’, Ultrasonics, 70. 10.1016/j.ultras.2016.05.001 (2016). 10.1016/j.ultras.2016.05.001 27179142

[33] Sharma, V. & Pandey, P. M. Recent advances in UAT: A step towards sustainability. *Cogent Eng.* **3** (1). 10.1080/23311916.2016.1222776 (2016).

[34] Sharma, V. & Pandey, P. M. ‘Experimental investigations and statistical modeling of surface roughness during ultrasonic-assisted turning with self-lubricating cutting inserts’, Proceedings of the Institution of Mechanical Engineers, Part E: Journal of Process Mechanical Engineering, 232(6). (2018). 10.1177/0954408917738127

[35] Sharma, V. & Pandey, P. M. Mechanistic based cutting force model during UAT with Self-Lubricating cutting inserts. *J. Adv. Manuf. Syst.* **18** (1). 10.1142/S0219686719500070 (2019).

[36] Singh, J., Singh, C. & Singh, K. ‘Rotary ultrasonic machining of advance materials: A review’, Materials Today: Proceedings. 10.1016/j.matpr.2023.01.159 (2023).

[37] Singh, R. & Khamba, J. S. Ultrasonic machining of titanium and its alloys: A review. *J. Mater. Process. Technol.* **173** (2). 10.1016/j.jmatprotec.2005.10.027 (2006).

[38] Sofuoğlu, M. A. et al. Experimental investigation of machining characteristics and chatter stability for Hastelloy-X with ultrasonic and hot turning. *Int. J. Adv. Manuf. Technol.* **95** (1–4). 10.1007/s00170-017-1153-9 (2018).

[39] Sofuoğlu, M. A. et al. Numerical investigation of hot UAT of aviation alloys. *J. Brazilian Soc. Mech. Sci. Eng.* **40** (3). 10.1007/s40430-018-1037-4 (2018).

[40] Soleimanimehr, H. Analysis of the cutting ratio and investigating its influence on the workpiece’s diametrical error in ultrasonic-vibration assisted turning. *Proc. Institution Mech. Eng. Part. B: J. Eng. Manuf.* **235** (4). 10.1177/0954405420968174 (2021).

[41] Tong, J., Zhao, B. & Jiao, F. Study on residual stress of GCr15 bearing steel under ultrasonic vibration turning. *Key Eng. Mater.*10.4028/www.scientific.net/KEM.522.173 (2012). 522.

[42] Umasekar, V. G. & John, M. R. S. Experimental investigations on UAT of magnesium silicon carbide. *Mater. Manuf. Processes*. **39** (5). 10.1080/10426914.2023.2289680 (2024).

[43] Umasekar, V. G. & Stalin John, M. R. ‘Experimental investigation on the machining performance of AISI D2 tool steel during ultrasonic-assisted turning under dry and MQL conditions’, Proceedings of the Institution of Mechanical Engineers, Part E: Journal of Process Mechanical Engineering, 237(6). 10.1177/09544089231190172 (2023).

[44] Venkata Sivareddy, D., Krishna, P. V. & Venu Gopal, A. ‘Effect of thermo-mechanical loading on machining induced residual stresses in ultrasonic vibration assisted turning of Ti6Al4V alloy’, Proceedings of the Institution of Mechanical Engineers, Part B: Journal of Engineering Manufacture, **236**(13). 10.1177/09544054221093565 (2022).

[45] Wang, H. et al. Surface grinding of carbon fiber-reinforced plastic composites using rotary ultrasonic machining: effects of tool variables. *Adv. Mech. Eng.* **8** (9). 10.1177/1687814016670284 (2016).

[46] Wang, H. et al. The effects of elliptical ultrasonic vibration in surface machining of CFRP composites using rotary ultrasonic machining. *Int. J. Adv. Manuf. Technol.* **106** (11–12). 10.1007/s00170-020-04976-w (2020).

[47] Wang, J. et al. Damage formation and suppression in rotary ultrasonic machining of hard and brittle materials: A critical review. *Ceram. Int.*10.1016/j.ceramint.2017.10.050 (2018).

[48] Wei, S. et al. ‘Study on surface roughness model of 3D ultrasonic vibration–assisted turning driven by a single actuator’, International Journal of Advanced Manufacturing Technology, **123**(11–12). 10.1007/s00170-022-10510-x (2022).

[49] Wei, S. et al. Theoretical and experimental study of 3D ultrasonic vibration-assisted turning driven by two actuators. *Measurement: J. Int. Meas. Confederation*. **215**10.1016/j.measurement.2023.112865 (2023).

[50] Xu, Y. et al. Experimental study on chip shape in ultrasonic vibration–assisted turning of 304 austenitic stainless steel. *Adv. Mech. Eng.* **11** (8). 10.1177/1687814019870896 (2019).

[51] Xu, Y. et al. Experimental study on cutting force in ultrasonic vibration-assisted turning of 304 austenitic stainless steel. *Proc. Institution Mech. Eng. Part. B: J. Eng. Manuf.* **235** (3). 10.1177/0954405420957127 (2021).

[52] Xu, Y. et al. Theoretical and experimental investigations of tool wear in ultrasonic vibration–assisted turning of 304 austenitic stainless steel. *Int. J. Adv. Manuf. Technol.* **127**, 7–8. 10.1007/s00170-023-11686-6 (2023).

[53] Ya, G. et al. ‘Analysis of the rotary ultrasonic machining mechanism’, Journal of Materials Processing Technology, **129**(1–3). 10.1016/S0924-0136(02)00638-6 (2002).

[54] Yu, Z. Y., Rajurkar, K. P. & Tandon, A. Study of 3D micro-ultrasonic machining. *J. Manuf. Sci. Eng.* **126** (4). 10.1115/1.1813482 (2004).

[55] Yuan, Z. et al. A comprehensive review of advances in ultrasonic vibration machining on SiCp/Al composites. *J. Mater. Res. Technol.*10.1016/j.jmrt.2023.04.245 (2023).

[56] Zamani, M., Farahnakian, M. & Elhami, S. ‘Employment of UAT in the fabrication of microtextures to improve the surface adhesion of the titanium implant’, Proceedings of the Institution of Mechanical Engineers, Part B: Journal of Engineering Manufacture, **235** (12). 10.1177/09544054211011029 (2021).

[57] Zhang, J. G. et al. Electromechanical dynamics model of ultrasonic transducer in ultrasonic machining based on equivalent circuit approach. *Sens. (Switzerland)*. **19** (6). 10.3390/s19061405 (2019). 10.3390/s19061405 PMC6470996 30901971

[58] Zhang, J. H. et al. Experimental study on Single⁃Excitation 3⁃D ultrasonic turning technology. *Dongbei Daxue Xuebao/Journal Northeastern Univ.* **44** (8). 10.12068/j.issn.1005-3026.2023.08.012 (2023).

[59] Zhang, S. et al. Design of a High-Speed rotary ultrasonic machining machine tool for machining microstructure of brittle materials. *Micromachines* **14** (8). 10.3390/mi14081544 (2023). 10.3390/mi14081544 PMC10456564 37630078

[60] Zhou, H. et al. Advances in rotary ultrasonic machining system for hard and brittle materials. *Adv. Mech. Eng.*10.1177/1687814019895929 (2019).

[61] Zou, L. et al. Investigation on diamond tool wear in ultrasonic vibration-assisted turning die steels. *Mater. Manuf. Processes*. **32** (13). 10.1080/10426914.2017.1291958 (2017).

[62] Zou, P. et al. Experimental investigation of ultrasonic vibration assisted turning of 304 austenitic stainless steel. *Shock Vib.* **2015**10.1155/2015/817598 (2015).

[63] Feizizadeh, B. et al. A GIS-based extended fuzzy multi-criteria evaluation for landslide susceptibility mapping. *Comput. Geosci.*10.1016/j.cageo.2014.08.001 (2014). 73. 26089577 10.1016/j.cageo.2014.08.001 PMC4376179

[64] Figueiredo, K. et al. Sustainable material choice for construction projects: A life cycle sustainability assessment framework based on BIM and Fuzzy-AHP. *Build. Environ.* **196**10.1016/j.buildenv.2021.107805 (2021).

[65] Frazão, T. D. C. et al. ‘Multicriteria decision analysis (MCDA) in health care: A systematic review of the main characteristics and methodological steps’, BMC medical informatics and decision making. (2018). 10.1186/s12911-018-0663-1 10.1186/s12911-018-0663-1 PMC6211490 30382826

[66] Frini, A. A multicriteria intelligence aid methodology using MCDA, artificial intelligence, and fuzzy sets theory. *Math. Probl. Eng.* **2017**10.1155/2017/9281321 (2017).

[67] Gigović, L. et al. GIS-Fuzzy DEMATEL MCDA model for the evaluation of the sites for ecotourism development: A case study of Dunavski ključ region, Serbia. *Land. Use Policy*. **58**10.1016/j.landusepol.2016.07.030 (2016).

[68] Indrajayanthan, V. et al. Investigation on current and prospective energy transition scenarios in Indian landscape using integrated SWOT-MCDA methodology. *Sustain. (Switzerland)*. **14** (9). 10.3390/su14094940 (2022).

[69] Ouma, Y. O. et al. ‘Optimization of urban highway bypass horizontal alignment: A methodological overview of intelligent spatial MCDA approach using fuzzy AHP and GIS’, Advances in Civil Engineering, 2014. (2014). 10.1155/2014/182568

[70] Thakur, P. et al. The group Decision-Making using pythagorean fuzzy entropy and the complex proportional assessment. *Sensors* **22** (13). 10.3390/s22134879 (2022). 10.3390/s22134879 PMC9269554 35808377

[71] Yatsalo, B. et al. Fuzzy extensions of PROMETHEE: models of different complexity with different ranking methods and their comparison. *Fuzzy Sets Syst.*10.1016/j.fss.2020.08.015 (2021). 422.

[72] Ziemba, P. Selection of electric vehicles for the needs of sustainable transport under conditions of uncertainty—a comparative study on fuzzy Mcda methods. *Energies* **14** (22). 10.3390/en14227786 (2021).

[73] Sha, X., Zhu, Y., Sha, X., Guan, Z. & Wang, S. ZHPO-LightXBoost: an integrated prediction model based on small samples for pesticide residues in crops. *Environ. Model. Softw.* **188**, 106440. 10.1016/j.envsoft.2025.106440 (2025).

[74] Sha, X., Si, X., Zhu, Y., Wang, S. & Zhao, Y. Automatic three-dimensional reconstruction of transparent objects with multiple optimization strategies under limited constraints. *Image Vis. Comput.* **160**, 105580. 10.1016/j.imavis.2025.105580 (2025).

[75] Ma, C. et al. High-efficiency topology optimization method for thermal-fluid problems in cooling jacket of high-speed motorized spindle. *Int. Commun. Heat Mass Transfer*. **169**, 109533. 10.1016/j.icheatmasstransfer.2025.109533 (2025).

[76] Liu, R. & Shen, W. Data acquisition of exercise and fitness pressure measurement based on artificial intelligence technology. *SLAS Technol.* **33**, 100328. 10.1016/j.slast.2025.100328 (2025). 40619065 10.1016/j.slast.2025.100328

[77] Liu, W. et al. Compensator-based fixed-time prescribed performance control of vehicular platoon with input nonlinearities: A performance boundary self-adjusting approach. *IEEE Trans. Intell. Transp. Syst.*10.1109/TITS.2025.3579923 (2025).

[78] Li, R. et al. Modeling and analysis of relative fatigue life under 3D mixed lubrication in marine helical gears. *Tribol. Int.* **211**, 110834. 10.1016/j.triboint.2025.110834 (2025).

[79] Ma, C. et al. Highly efficient heat dissipation method of grooved heat pipe for thermal behavior regulation for spindle system working in low rotational speed. *Int. Commun. Heat Mass Transfer*. **169**, 109575. 10.1016/j.icheatmasstransfer.2025.109575 (2025).

[80] Ni, Z. L. et al. Numerical analysis of ultrasonic spot welding of Cu/Cu joints. *J. Mater. Eng. Perform.* **34**, 20624–20635. 10.1007/s11665-025-10733-5 (2025).

[81] Xu, J. et al. Study on fuel injection stability improvement in marine low-speed dual-fuel engines. *Appl. Therm. Eng.* **253**, 123729. 10.1016/j.applthermaleng.2024.123729 (2024).

[82] Liu, Z. et al. Effect of lateral stress and loading paths on direct shear strength and fracture of granite under true triaxial stress state by a self-developed device. *Eng. Fract. Mech.* **318**, 110952. 10.1016/j.engfracmech.2025.110952 (2025).

[83] Laghari, R. A., Li, J., Laghari, A. A. & Wang, S. Q. A review on application of soft computing techniques in machining of particle reinforcement metal matrix composites. *Arch. Comput. Methods Eng.* **27** (5), 1363–1377. 10.1007/s11831-019-09340-0 (2019).

[84] Ali, M. A. et al. Effects of turning parameters and parametric optimization of the cutting forces in machining SiCp/Al 45 wt% composite. *Metals* **10** (6), 840. 10.3390/met10060840 (2022).

[85] Laghari, R. A. & Sarhan, A. A. D. Parametric modelling and optimization for machinability performance enhancement of difficult-to-cut SiCp/Al (0%) MMCs using ANFIS and MRA. *Int. J. Interact. Des. Manuf. (IJIDeM)*. **19**, 7315–7337. 10.1007/s12008-025-02282-x (2025).

[86] Laghari, R. A., He, N., Jamil, M. & Gupta, M. K. Tribological and machining characteristics of milling SiCp/Al MMC composites under sustainable cooling conditions. *Int. J. Adv. Manuf. Technol.* **128**, 2613–2630. 10.1007/s00170-023-12083-9 (2023).

[87] Laghari, R. A., Pourmostaghimi, V., Laghari, A. A., Qazani, M. R. & Sarhan, A. A. D. Genetic modeling for enhancing machining performance of High-Volume fraction 45% SiCp/Al particle reinforcement metal matrix composite. *Arab. J. Sci. Eng.* **50**, 9043–9059. 10.1007/s13369-024-09330-w (2024).

[88] Sundar Singh Sivam, S. P., Harshavardhana, N. & Kumaran, D. Intuitionistic fuzzy TOPSIS-based optimization of microcups production from directionally rolled copper strips for sustainability. *Multiscale Multidiscip Model. Exp. Des.* **7**, 4819–4832. 10.1007/s41939-024-00479-3 (2024).

[89] Sundar Singh Sivam, S. P., Harshavardhana, N. & Rajendran, R. Sustainability-driven optimization of directionally rolled copper strips for deep drawing microcups through response surface methodology. *J. Braz Soc. Mech. Sci. Eng.* **45**, 632. 10.1007/s40430-023-04543-w (2023).

[90] Sundar Singh, S. P. & Sivam, Rajendran, R. A study on deep drawn cups and selecting optimal settings deploying ANN training and architectural parameters using the Taguchi ARAS approach. Proceedings of the Institution of Mechanical Engineers, Part C: Journal of Mechanical Engineering Science. 0(0). 10.1177/09544062221135532 (2022). IF 1.758.

[91] Sundar Singh, S. P. & Sivam, Rajendran, R. Hybrid optimisation of input process parameters of deep-drawn cylindrical cups from directional rolled copper strips. Proceedings of the Institution of Mechanical Engineers, Part C: Journal of Mechanical Engineering Science. 0(0). 10.1177/09544062221137198 (2022), IF 1.758.

[92] Sundar Singh Sivam, S. P., Rajendran, R. & Harshavardhana, N. An investigation of hybrid models FEA coupled with AHP-ELECTRE, RSM-GA, and ANN-GA into the process parameter optimization of high-quality deep-drawn cylindrical copper cups. *Mech. Based Des. Struct. Mach.*10.1080/15397734.2022.2120497 (2022).

